# Reaction Environment Design for Multigram Synthesis via Sonogashira Coupling over Heterogeneous Palladium Single-Atom Catalysts

**DOI:** 10.1021/acssuschemeng.3c04183

**Published:** 2023-11-22

**Authors:** Dario Poier, Dario Faust Akl, Elysia Lucas, Alicia Rodrigues Machado, Georgios Giannakakis, Sharon Mitchell, Gonzalo Guillén-Gosálbez, Roger Marti, Javier Pérez-Ramírez

**Affiliations:** † Institute of Chemical Technology, Haute école d’ingénierie et d’architecture Fribourg, HES-SO University of Applied Sciences and Arts Western Switzerland, Fribourg 1700, Switzerland; ‡ Institute for Chemical and Bioengineering, Department of Chemistry and Applied Biosciences, ETH Zurich, Zurich 8093, Switzerland

**Keywords:** cross-coupling, single-atom heterogeneous catalyst, active pharmaceutical ingredient, scale-up, life-cycle assessment

## Abstract

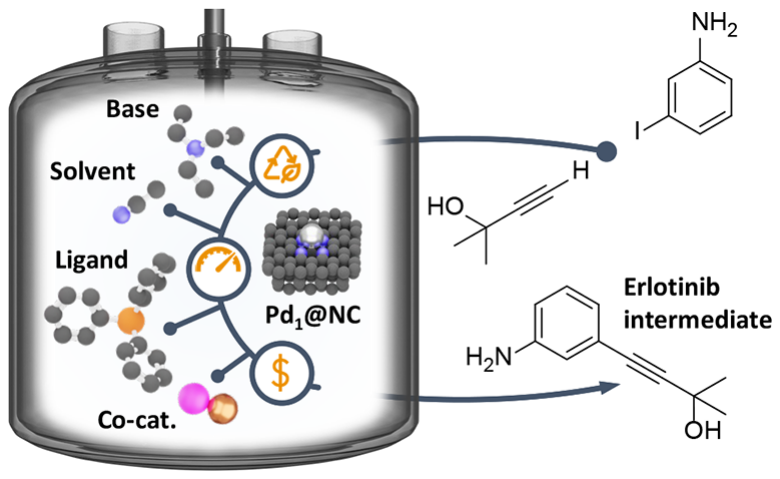

Single-atom heterogeneous catalysts (SACs) attract growing interest in their application in green chemistry and organic synthesis due to their potential for achieving atomic-level precision. These catalysts offer the possibility of achieving selectivity comparable to the traditionally applied organometallic complexes, while enhancing metal utilization and recovery. However, an understanding of SAC performance in organic reactions remains limited to model substrates, and their application as drop-in solutions may not yield optimal activity. Here, we investigate the previously unaddressed influence of the reaction environment, including solvent, base, cocatalyst, and ligand, on the performance of a palladium SAC in Sonogashira–Hagihara cross-couplings. By examining the effects of different solvents using the established criteria, we find that the behavior of the SAC deviates from trends observed with homogeneous catalysts, indicating a distinct interplay between heterogeneous systems and the reaction environment. Our results illustrate the satisfactory performance of SACs in cross-couplings of aryl iodides and acetylenes with electron-withdrawing and -donating groups, while the use of bromides and chlorides remains challenging. Extending the proof-of-concept stage to multigram scale, we demonstrate the synthesis of an intermediate of the anticancer drug Erlotinib. The catalyst exhibits high stability, allowing for multiple reuses, even under noninert conditions. Life-cycle assessment guides the upscaling of the catalyst preparation and quantifies the potential environmental and financial benefits of using the SAC, while also revealing the negligible impact of the PPh_3_ ligand and CuI cocatalyst. Our results underscore the significant potential of SACs to revolutionize sustainable organic chemistry and highlight the need for further understanding the distinct interplay between their performance and the reaction environment.

## Abstract 2

Reaction design study reveals environmental benefits of employing single-atom heterogeneous catalysts in Sonogashira-Hagihara cross-coupling, which are quantified through life-cycle assessment.

## Introduction

Since the breakthroughs in homogeneous transition metal catalysis in the 1970s, methodologies such as the Mizoroki–Heck, Sonogashira–Hagihara, or Suzuki–Miyaura reactions have gained significant attention.^1−4^ These reactions enable atom-efficient formation of carbon–carbon bonds under mild conditions, while exhibiting remarkable tolerance toward various functional groups through appropriate catalyst design, and find broad applications in the production of fine chemicals.^5−7^ However, despite decades of research dedicated to improving the efficiency of organometallic catalysts and reaction conditions, several challenges persist in their practical application. These include issues with the stability under noninert conditions, the expense of metals and ligands, difficulties in transitioning from batch to continuous flow, and catalyst separation and metal recovery postreaction.^8,9^

In response to these challenges, researchers have explored alternative approaches, such as the replacement of organometallic catalysts with heterogeneous alternatives. Single-atom heterogeneous catalysts (SACs) have recently emerged as a promising approach to address the challenges of fine chemical synthesis.^8,10^ Unlike conventional, i.e., nanoparticle-based, heterogeneous catalysts, SACs offer superior control over the coordination sphere of active centers and the advantage of easy recyclability, combining the atomically precise and atom-efficient nature of homogeneous catalysts with the reusability of heterogeneous analogs.^11−16^ In a previous study, a palladium SAC (Pd_1_@NC) demonstrated comparable yields to homogeneous benchmarks in the Sonogashira–Hagihara cross-coupling (herein referred to as Sonogashira coupling), which is a prominent C–C bond forming reaction in the pharmaceutical industry, for introducing alkynyl moieties through the C*sp*^2^–C*sp* bond formation.^7,17−19^ The SAC exhibited slower kinetics compared to the homogeneous palladium acetate system but achieved comparable selectivity. An ex-ante life-cycle assessment (LCA) revealed the major potential sustainability benefits the SAC could bring assuming that it could be reused in just a few cycles.^20^ However, the current literature lacks systematic studies on the behavior of SACs in different chemical environments, which is surprising considering the substantial effect the choice of reaction components (solvents, bases, ligands, cocatalysts, and other additives) is known to have on the performance of homogeneous catalysts. Furthermore, most previous studies have tackled simple model compounds at a small scale.

Here, we study the impact of various reaction parameters on the performance of Pd_1_@NC in the Sonogashira coupling and evaluate their potential for improving sustainability and reducing costs when applied at the large scale. Using a prototypical Sonogashira coupling at first, we uncover distinct deviations from trends observed for homogeneous catalysts by systematically examining the effects of the solvent, base, ligand, and cocatalyst. Subsequently, the practical potential of the Pd_1_@NC catalyst is demonstrated through successful upscaling of its preparation and application in the multigram synthesis of an intermediate of the anticancer drug Erlotinib.^18,21,22^ LCA guides the catalyst and reaction optimization at scale, providing insights into the potential environmental and economic benefits. The application of SACs to the synthesis of industrially relevant target molecules demonstrates the practical potential of these heterogeneous catalysts as replacements for homogeneous systems. The insights obtained from this study present a pivotal first step in advancing our understanding of the interactions between the supported palladium single atoms and the chemical environment and its impact on the catalyst performance, as well as sustainability. This will not only aid the further development of SAC for the Sonogashira cross-coupling but also lay the foundation for harnessing the immense potential of these materials for the sustainable heterogeneously catalyzed fine chemical synthesis.

## Materials and Methods

Nitric acid (>65 wt %, puriss.) and dicyandiamide (99%) were purchased from Sigma-Aldrich, activated carbon (AC, Norit Rox 0.8) from Cabot Corporation, (NH_3_)_4_Pd(NO_3_)_2_ (5 wt % water), and Pd(NO_3_)_2_·2H_2_O (41 wt % Pd) from abcr. The reagents for the cross-coupling reactions were purchased from Chemie Brunschwig AG. All chemicals were used without further purification.

### Catalyst Preparation

As the first step of nitrogen incorporation, AC (23.0 g) was sieved (sieve fraction <0.2 mm) and refluxed in nitric acid (4 M, 0.46 dm^3^) at 353 K for 16 h. The mixture was poured into DI water (273 K, 0.50 dm^3^), filtered, washed copiously with DI water (4.6 dm^3^), and dried overnight (338 K). The acid-AC was added to a solution of dicyandiamide (69.0 g, 0.82 mol) in acetone (0.69 dm^3^), which was subsequently evaporated at 353 K under constant stirring. Finally, the dried solid was gently crushed, transferred to ceramic boats, and carbonized in flowing nitrogen (723 K, 3 h hold, then 923 K, all ramps 5 K min^–1^) to obtain nitrogen-doped carbon (NC, 55.2 g) as a black powder. (NH_3_)_4_Pd(NO_3_)_2_ (5.00 mg, 0.02 mmol) and DI water (15 cm^3^) were added to a sonicated (30 min) suspension of NC (1 g) in DI water (20 cm^3^) and vigorously stirred overnight. The suspension was then subjected to repeated cycles (20) of microwave irradiation (100 W for 15 s) with pressurized air cooling (45 s between cycles), maintaining a sample temperature of 30 °C. Afterward, the solid was separated through centrifugation, washed with water (5 × 5 cm^3^) and ethanol (5 × 5 cm^3^), dried overnight (338 K), and annealed in a tubular oven under nitrogen flow (573 K, 5 h hold, 5 K min^–1^ ramp) to obtain the Pd_1_@NC (1 g) as a black solid. A detailed procedure for the large-scale preparation of Pd_1_@NC can be found in the Supporting Information.

### Catalyst Characterization

The thermogravimetric analysis (TGA) was carried out on a Linseis STA PT 1600 instrument using an alumina crucible. The sample (20 mg) was dried (373 K, 1.5 h) and subsequently heated to 923 K (5 K min^–1^) under an inert atmosphere (300 cm^3^ min^–1^, 6.6 vol % He in Ar). The metal content was analyzed by the inductively coupled plasma optical emission spectroscopy (ICP-OES) using a Horiba Ultra 2 instrument (photomultiplier tube detector). Sample aliquots (15 mg) were subjected to a microwave digestion treatment (473 K, 20 min, 48 bar) using concentrated nitric acid (>65 wt %, 3 cm^3^) to dissolve the matrix. Liquid organic samples were dried (338 K) and mineralized with a mixture of hydrogen peroxide (1 cm^3^) and sulfuric acid (>95%, 3 cm^3^). The obtained solutions were diluted with Milli-Q water, and solids were removed through polytetrafluoroethylene (PTFE) syringe filters (0.25 μm pore size). Argon sorption (77 K) was conducted with a Micromeritics TriFlex analyzer over previously degassed specimens (8 h, 423 K, vacuum). For scanning transmission electron microscopy (STEM), the powder samples were dusted onto carbon-film copper and nickel grids (300 mesh). High-angle annular dark-field (HAADF) STEM and energy dispersive X-ray spectroscopy (EDX) were performed on a FEI Talos F200X microscope with a SuperX detector (200 kV acceleration potential). EDX elemental maps were averaged over five frames (1024 × 1024 pixels, 15 ms pixel dwell time) in the spectral range up to 20 keV and postprocessed (background subtraction and Gaussian blur). Aberration corrected annular dark-field STEM (AC-ADF-STEM) images of the as-prepared and used catalysts were acquired on a Hitachi HD-2700CS instrument operated at 200 kV. X-ray photoelectron spectroscopy (XPS) was conducted on a Physical Electronics Instruments Quantum 2000 instrument with monochromatic Al Kα radiation (15 kV, 32.3 W). The spectral acquisition occurred under ultrahigh vacuum conditions (5 × 10^–8^ Pa residual pressure) with a pass energy of 46.95 eV. All XPS signals were referenced using the C1*s* photoemission, which was set at 284.8 eV. Peak fitting with the reference spectra^23−25^ was conducted using the CasaXPS software.^26^ X-ray absorption spectroscopy (XAS) was conducted at the X10DA (SuperXAS) beamline of the Swiss Light Source. The X-ray beam from the 2.9 T superbend was collimated using a Pt-coated mirror, monochromatized using a Si(111) channel-cut monochromator, and focused to a spot size of 500 × 100 μm^2^ (horizontal × vertical) using a Pt-coated toroidal mirror. Data were acquired from pressed pellets at the Pd K-edge in the transmission mode, using three 15 cm long Ar/N_2_-filled ionization chambers. The samples were placed between the first and second ionization chambers. For the absolute energy calibration, the palladium foil was measured simultaneously between the second and third ionization chambers. The resulting spectra were energy calibrated, background corrected, and normalized using the Athena program from the Demeter software suite.^27^ Characterization of the as-prepared Pd_1_@NC and after its application in the Sonogashira coupling can be found in the Supporting Information (Figure S2–S5 and Table S14).

### Catalyst Evaluation

Unless otherwise stated, the Sonogashira coupling reaction was performed following a standard procedure: a degassed solution consisting of halide (1 equivalent, equiv), alkyne (1.1 equiv), base (2.2 equiv), 1,3,5-trimethylbenzene (0.25 equiv, internal standard), and solvent (0.5 M) was added to the palladium catalyst (0.5 wt % Pd, 0.1 mol %), copper(I) iodide (CuI, 1 mol %), and triphenylphosphine (PPh_3_, 1 mol %) and vigorously stirred for 24 h at 353 K under a protective atmosphere (Ar). After cooling to room temperature, the SAC was separated from the reaction mixture by filtration. The reaction solution was analyzed by gas chromatography flame ionization detection (GC-FID).

To simplify the workflow for condition screenings, stock solutions consisting of aryl halide, acetylene, solvent, base, and internal standard were prepared whenever reasonable. If properly degassed by at least three freeze-pump-thaw cycles and stored under an inert atmosphere afterward, the stock solutions could be stored for multiple weeks without any change in the composition. This was monitored by GC-FID, and a reference (*t* = 0) sample was taken before the use of the respective stock solution as comparison to the postreaction analysis. The GC-FID was performed on a Thermo TRACE 1300 chromatograph equipped with a flame ionization detector and a ZB-5 column (5%-phenyl-95%-dimethylpolysiloxane, 30 m length, 0.25 mm inner diameter, 0.25 μm film thickness) using helium as a carrier gas. Nuclear magnetic resonance (NMR) spectra were recorded with a Bruker 300 Ultrashield spectrometer and referenced against the chemical shift of the residual protio-solvent peak (CDCl_3_: 7.26 ppm; DMSO-d_6_: 2.50 ppm) for ^1^H NMR and the deuterated solvent peak (CDCl_3_: 77 ppm; DMSO-d_6_: 40 ppm) for ^13^C NMR measurements. The full solvent and base screening results, protocols for the 50 cm^3^ recycling, and the large-scale application, as well as the NMR spectra and signal documentation of all products are reported in the Supporting Information (Tables S1 and S2 and Figures S9–S15).

### Life-Cycle Assessment

The process that was developed in this work was subjected to an LCA to determine its potential environmental impact if scaled up to an industrial level, as well as to compare the contributions from various process aspects. For this purpose, an ex-ante cradle-to-gate assessment was performed in accordance with the principles in the ISO 14040:2006 standards^28^ and following guidelines for the evaluation of laboratory scale chemical processes in LCA studies.^29^ This analysis considers all upstream activities associated with the material and energy inputs for the synthesis of intermediate **13**, as well as the downstream processing of major waste streams. The foreground system was modeled using the experimental data, while the background system (upstream and downstream activities) utilized data from the ecoinvent database v3.9.^30,31^ The total global warming potential (GWP) associated with the synthesis was estimated using the IPCC 2013 GWP (100-year time horizon) life-cycle impact assessment method, expressed in terms of the amounts of equivalent kilograms of carbon dioxide equivalents emitted per kg of **13** (kg_CO2-equiv_ kg**_13_**^–1^). The total impact encompasses contributions from the catalytic system (Cat. System; Pd-catalyst, cocatalyst, and ligand), reagents (coupling reagents), solvent mixtures (solvent and base), and the classes’ energy (heat and electricity for the process operation), and waste (incineration of the solvent mixture). To approximate the impact of Pd_1_@NC on the process cost and carbon footprint, the GWP of the synthesis energy consumption and source materials were considered. It was assumed that the catalyst can be used 10 times before the end of its usable lifetime is reached, and the palladium is recovered, refined, and used to resynthesize the catalyst; however, recovery and refinery methods are omitted at this point. Based on the previous work, a Pd recovery rate of 98% is expected at the end of the usable lifetime,^20^ while assuming no Pd loss during the catalyst’s application. Contributions originating from the energy class, comprising demands for heating, stirring, and filtration, are calculated for a 100 dm^3^ batch reactor according to the literature. Although the recovery of CuI and recycling of the solvent mixture would be possible, here, it is deemed waste after completion of the reaction and considered to be incinerated after the product and catalyst separation. The resultant GWP estimate from this work is therefore likely higher than an estimate from a more detailed assessment using a further optimized system. Further information, including a detailed description of the LCA framework and uncertainty calculation, the source materials assumed in the upstream analysis and reagent costs (Schemes S3 and S4 and Tables S3–S11), Pd_1_@NC and Erlotinib intermediate **13** cost and GWP calculations (Table S12 and S13), and the detailed upstream analysis of the top GWP contributors in the synthesis of intermediate **13** (Figure S8) can be found in the Supporting Information.

## Results and Discussion

### Multigram Preparation of Pd_1_@NC

**Figure 1:**
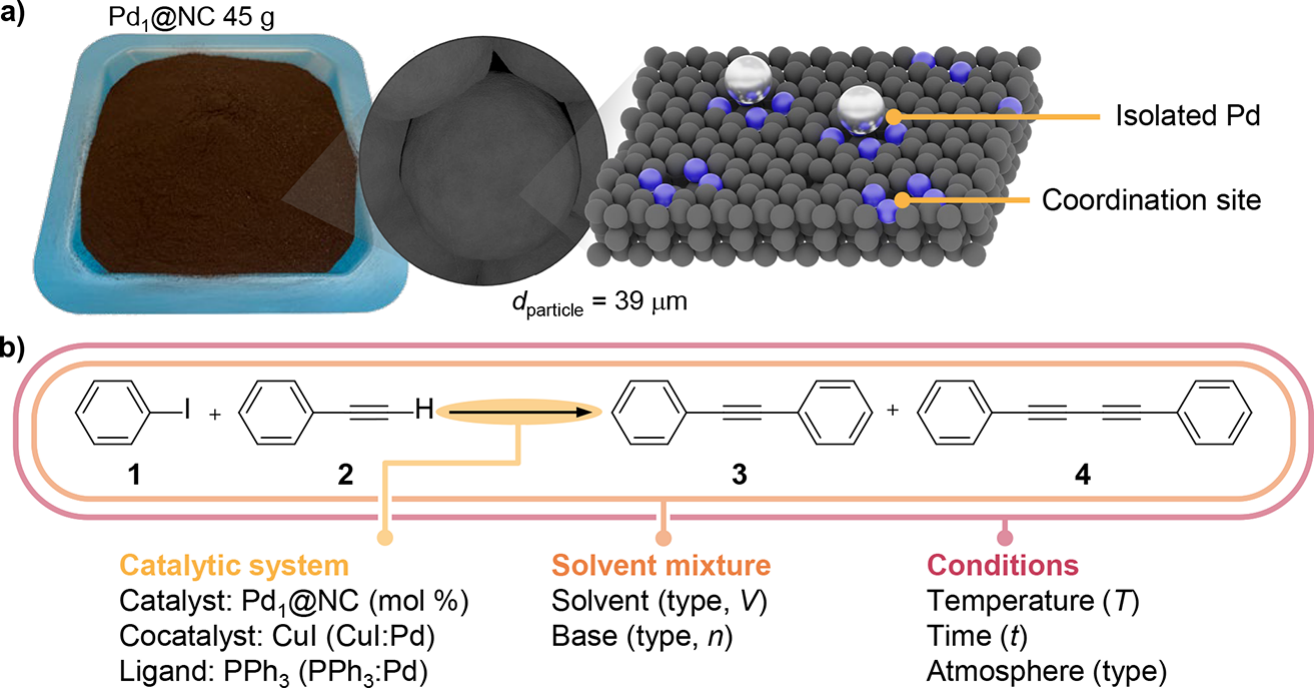
(a) Photograph of the large batch of the Pd_1_@NC catalyst and schematic of its structure at the particle and nanometer scale. Color code: blue, nitrogen; black, carbon; gray, palladium. (b) Reaction scheme of the Sonogashira coupling of iodobenzene (**1**) and phenylacetylene **2** toward diphenylacetylene **3** and the undesired Glaser homocoupling product **4**. The parameter of the catalytic system (yellow), solvent mixture (orange), and exterior conditions (red) that were investigated in this work are shown in parenthesis.

The choice of the Pd_1_@NC (Figure 1a) was based on the previous work, assessing the general environmental advantage of the SAC over organometallic catalysts applied in the Sonogashira coupling by the cradle-to-gate LCA.^20^ To determine its potential for large-scale applications, the possibility for optimizations in the catalyst preparation protocol was evaluated. The first step involves the preparation of the NC carrier via nitrogen-doping of an activated carbon AC. The previous protocol employed a two-step temperature program, with a 3 h treatment at 723 K and a 2 h treatment at 923 K. However, thermogravimetric analysis (TGA) of the AC with the nitrogen precursor shows that the mass loss ceases already during the heating phase toward 923 K (Figure S1), revealing the potential to avoid over 60% of the whole treatment’s energy consumption (4.67 kWh) by stopping the treatment after reaching the desired temperature, as quantified by the LCA. The metal deposition and the catalyst post-treatment were also optimized. While the initial procedure involved the deposition of the palladium precursor in aqua regia for the deposition, the use of water as the deposition medium was also found to be effective microwave irradiation, which is usually used to achieve high metal dispersion and avoid nanoparticle formation.^32^ Similarly, it was possible to omit the subsequent preparation of the Pd_1_@NC via standard wet impregnation of the palladium precursor onto the carrier yielded a material exhibiting atomically dispersed metal species and no nanoparticles. Furthermore, it was observed that washing the catalyst with water after the deposition is sufficient, simplifying the preparation and improving the environmental footprint, by avoiding the use of additional organic solvents. Also, similar catalyst quality was obtained when the annealing of the material was performed in static rather than flowing nitrogen atmosphere, significantly decreasing the gas consumption. With an adapted protocol, 50 g of Pd_1_@NC could be prepared in one batch, exhibiting equivalent properties to the previously reported samples. HAADF-STEM imaging of the as-prepared catalyst confirmed the isolated nature of the palladium centers through the absence of any metal aggregates, while EDX maps evidenced a uniform dispersion of the metal across the carrier material (Figure S2). A palladium content of 0.49 wt % was determined by the ICP-OES, and XANES and XPS analyses verified the palladium coordination as positively charged species (Pd^2+^), consistent with the properties obtained from the previous smaller scale syntheses (Figure S3). It can be stored under ambient conditions over extended periods of time without the need to ensure oxygen or moisture free conditions to preserve its activity and structural properties.

The Sonogashira coupling of iodobenzene (**1**) and phenylacetylene **2** (Figure 1b) was used as a prototypical reaction to initially explore the impact of reaction environments on the SAC performance. It presents the simplest set of coupling partners for this type of reaction and as such permits the investigation of parameter effects without superimposing substituent-induced structural or electronic effects. It also facilitates the comparison of the obtained results with the literature data.

### Influence of the Reaction Environment

**Figure 2:**
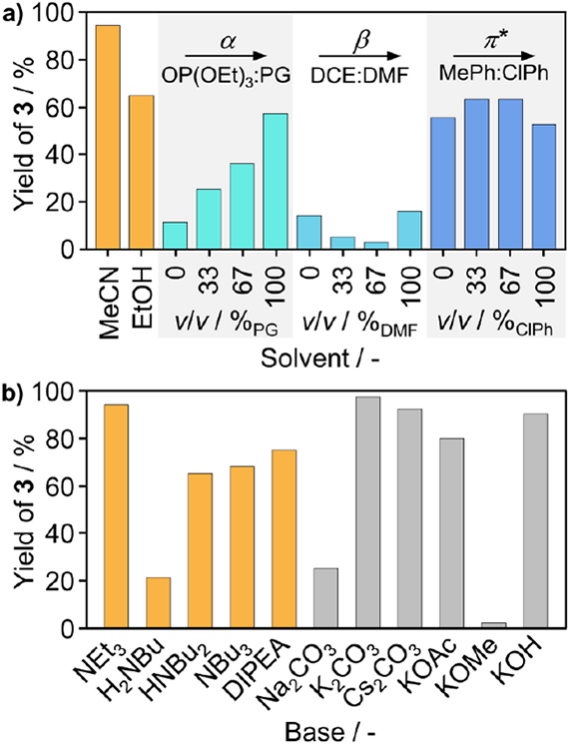
Effect of varying the (a) solvent or (b) base on the yield of alkynyl **3**. The solvents (except for MeCN and EtOH) were chosen as the triethylphosphate:propane-1,2-diol (OP(OEt)_3_:PG, propylene glycol), dimethylformamide:1,2-dichloroethane (DMF:DCE), and toluene:chlorobenzene (MePh:ClPh) solvent pairs, which mostly differ in their proticity α, basicity β, and polarizability/polarity π* (all ranging from 0 to 1). The solvent mixtures (neat solvent, 2:1, 1:2) are arranged in the order of ascending parameter value. Standard conditions: iodobenzene (**1**, 1 equiv), phenylacetylene **2** (1.5 equiv), NEt_3_ (2.2 equiv), MeCN (0.4 M), Pd_1_@NC (0.5 wt % Pd, 0.2 mol %), CuI (2 mol %), PPh_3_ (1 mol %), and trimethylbenzene (0.125 M) as the internal standard, at 353 K, 24 h, under Ar. Yields were determined by GC-FID.

Solvents, which often comprise the largest proportion of the reaction mixture, are crucial in the chemical process design. Their proper selection serves a dual role, ensuring safety and minimizing the environmental impact while also influencing the reaction environment, including the stabilization of the intermediate species and reaction products, affecting the reaction kinetics and selectivity.^33,34^ In an effort to transform the categorical variable of solvent type into a continuous parameter, the impact of solvent-specific properties^35^ on catalytic performance was systematically assessed. To this end, specific solvent pairs were selected to probe the effect of each Kamlet–Taft parameter: proticity α, basicity β, and polarizability-polarity π* (all three ranging from 0 to 1).^36^ The solvents were tested in the pure form as well as in 2:1 and 1:2 mixtures, thus resulting in a comprehensive map of the parameter space. To avoid solubility issues affecting the determined productivity of the catalyst, triethylamine (NEt_3_) was chosen as the base for the study of solvent properties. It exhibits good solubility in a variety of solvents and can be classified as a weak nucleophile, which avoids strong metal–base interactions that could superimpose the effect of solvent properties. Comparison of the yield of **3** observed after 24 h (Figure 2a) revealed significant differences using propane-1,2-diole (propylene glycol, PG, 57%, α: 0.83) as a solvent compared to triethylphosphate (OP(OEt_3_), 11%, α: 0.00), suggesting a strong impact of the proticity. In contrast, variation of the β or π* parameters did not show an obvious correlation with the yield. Interestingly, toluene and chlorobenzene, which have a reported α value of 0.00, yielded similar results as the dedicated α pair. Additionally, acetonitrile (MeCN) with similar π* and a smaller α value than the 2:1 mixture of triethylphosphate:1,2-propanediol outperformed the latter system.

Surprisingly, acetonitrile, ethanol, and toluene exhibited an overall good performance, while low yields were observed in dimethylformamide (DMF). This is unexpected considering that DMF has been widely used in homogeneously catalyzed cross-coupling in the past.^33,35^ A study that experienced low activity (far below 10% yield) in DMF for the homogeneously catalyzed Sonogashira coupling as well, using conditions similar to this work, found the same for MeCN and ethanol and only moderate activity for toluene.^37^ Other work, however, claimed that only a combination of moderately polar aprotic solvent with an inorganic base (MeCN, Cs_2_CO_3_) would yield satisfactory results.^38^ Similar inconsistent observations have been reported for heterogeneous nanoparticle-based catalysts supported on silica,^39^ polyaniline,^40^ or metal organic framework.^41^ A possible explanation for the unexpected low activity could be a strong interaction between the catalytically active center and the solvent molecule,^35^ leading to a competitive coordination between the solvent and reactants, effectively poisoning the catalyst. These observations highlight the complex role that the solvent inherits, acting as a medium for the reagent dissolution and transfer, as well as interacting with the active center and intermediates. This clearly emphasizes the importance of assessing the impact of the solvent mixture when developing new catalytic systems.

The choice of the base is crucial, as well as it is responsible for the deprotonation of the terminal ethyne moiety. A range of organic and inorganic bases was explored to identify the most effective candidates in MeCN as the solvent. Variation of the degree of substitution and steric hindrance of the amine base (Figure 2b) did not improve the product yields compared to NEt_3_. Amines substituted to a higher degree (HNBu_2_, NBu_3_) generally performed better than primary amines (H_2_NBu), consistent with their higher basicity, thus facilitating better acetylene deprotonation. *N*,*N*-Diisopropylethylamine (DIPEA, 75%) albeit exhibiting higher basicity and being structurally similar to triethylamine shows lower activity. As amines are known to demonstrate ligandlike behavior and support active species in the catalytic cycle, this could indicate an amine–metal center interaction that is prevented in the case of DIPEA due to its great steric demand.^42^ Looking at the class of inorganic bases, only a moderate yield was obtained with sodium carbonate (Na_2_CO_3_, 26%), while the use of carbonates accompanied by bigger counter cations (i.e., potassium, 97% and cesium, 92%) led to significant higher yields. Among different potassium salts, the carbonate was found to be the best choice as the counteranion, although the difference to the hydroxide (KOMe, 80%) and acetate (KOH, 89%) was small. It should be noted that the base selection is strongly influenced by the base–solvent interaction, particularly in the case of using an apolar solvent like toluene in combination with inorganic carbonates where, for example, the solubility constraints can impact the observed yield.^39^ Although yields obtained with K_2_CO_3_ were slightly higher than those with NEt_3_, the tertiary amine was selected to continue with the API synthesis due to the desire to achieve a homogeneous reaction solution that contains only the catalyst as an insoluble component, which facilitates the separation and characterization of the catalyst after the reaction.

**Figure 3:**
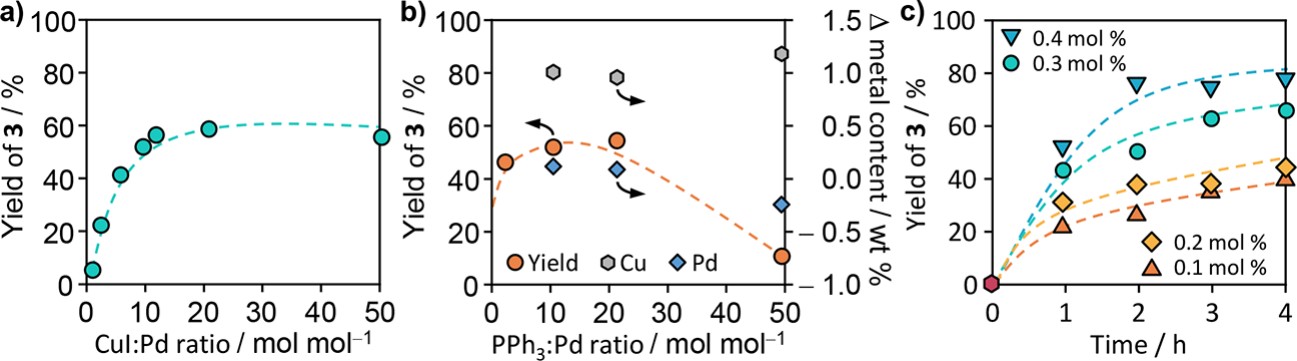
Effect of the (a) CuI:Pd and (b) PPh_3_:Pd ratios with the postreaction determined change in the palladium (blue diamond) and copper (pink hexagon) content of the Pd_1_@NC corresponding to the axis on the right-hand site. Arrows indicate the axis applicable to the data set. (c) Catalyst amount (inset indicated as mol % Pd) on the yield of alkynyl **3**. Standard conditions unless specified otherwise: iodobenzene (**1**, 1 equiv), phenylacetylene **2** (1.5 equiv), NEt_3_ (2.2 equiv), MeCN (0.4 M), Pd_1_@NC (0.5 wt % Pd, 0.1 mol %), CuI (2 mol %), PPh_3_ (1 mol %), and trimethylbenzene (0.125 M) as the internal standard, at 353 K, 24 h, under Ar. Yields were determined by the GC-FID.

In the Sonogashira coupling, both CuI and PPh_3_ play pivotal roles. CuI acts as a cocatalyst, facilitating the activation of the alkyne and promoting the transmetalation step,^43^ by forming a reactive copper acetylide intermediate. While the use of other copper(I) salts or even copper flow-reactors that continuously release copper(I) ions into reaction solution is also possible,^44^ here, we focus on the commonly utilized CuI to aid reproducibility and limit the number of anionic species in the reaction environment. The presence of the cocatalyst becomes especially important, considering that the active centers on the Pd_1_@NC are spatially isolated as well as immobilized, which prevents a tandem Pd/Pd mechanism.^45^ PPh_3_, however, is hypothesized to serve as a metal coordinating phosphine ligand, enhancing the catalytic activity and influencing the reaction kinetics. To evaluate the activity contributions of the individual reaction components, reference experiments were conducted demonstrating negligible activity toward product **3** (<11% after 48 h) unless in combination with Pd_1_@NC (Figure S6). Under oxygen free conditions, the beneficial effect of CuI on the reaction kinetics reaches a maximum at a molar ratio of 20:1 CuI:Pd (Figure 3a). Above that ratio, the excess of activated alkyne limits the number of Pd^II^ centers available for the transmetalation. The observed beneficial effect of PPh_3_ plateaus above a PPh_3_:Pd molar ratio of 5:1 and diminishes beyond one of 10:1 (Figure 3b). Notably, the amount of PPh_3_ used in the heterogeneously catalyzed reaction is higher than the typically reported optimal amounts of monodentate phosphine ligands for organometallic catalysts that range from 2:1 up to 4:1. A possible explanation for the higher amount of PPh_3_ required could be to compensate for the ligand oxidation. Handling the SAC under noninert conditions prior to the reaction introduces some oxygen into the system, leading to the oxidation of PPh_3_ to OPPh_3_, which does not promote the catalyst performance.^46^ Even under strictly inert conditions, including the prolonged storage of all solids under a vacuum before adding a degassed mixture of all liquid components, the GC-FID analysis showed the formation of OPPh_3_ and the Glaser homocoupling product **4** (2%). The decrease in performance beyond a 10:1 ratio may be attributed to a competitive coordination of the ligand and substrates to the metal center. With increasing amounts of the catalytic system, a steady increase in activity was observed within a range of 0.1 to 0.4 mol % Pd (keeping the Pd:CuI:PPh_3_ molar ratio constant, Figure 3c and S6). The reaction temperature was found to play a crucial role as well, as an exponential increase in activity was observed within a range of 293–353 K, accompanied by a decrease in Glaser product **4** formation (Figure S6). Based on the temperature dependency, an apparent activation energy *E*_a_ of about 67 kJ mol^–1^ was determined using the Arrhenius equation (Figure S6).

### Functional Group Tolerance

**Figure 4:**
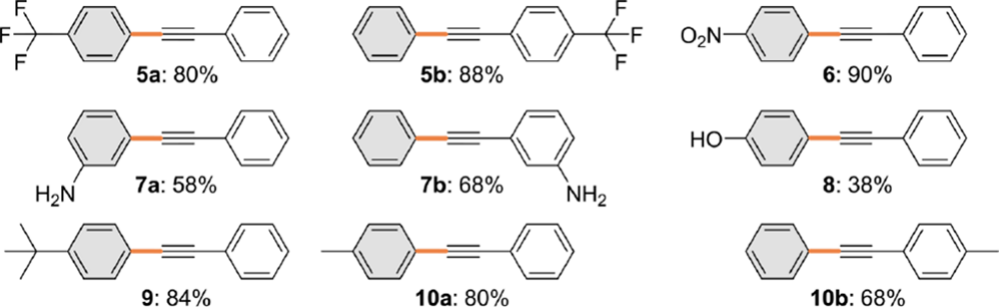
Product yields obtained in the Pd_1_@NC-catalyzed Sonogashira coupling, showcasing the scope of accessible products with the newly formed bond drawn in orange and the employed aryl halide colored in gray. Conditions: aryl halide (1 equiv), alkyne (1.5 equiv), NEt_3_ (2.2 equiv), MeCN (0.4 M), Pd_1_@NC (0.5 wt % Pd, 0.2 mol %), CuI (2 mol %), PPh_3_ (1 mol %), and trimethylbenzene (0.125 M) as the internal standard, at 353 K, 24 h, under Ar. Yields were determined by the GC-FID.

To assess the robustness of the catalyst, we investigated substituent-induced effects on the Pd_1_@NC activity through the coupling of various substituted aryl halides and alkynes. The coupling of bromo- and chlorobenzene with phenylacetylene resulted in significantly lower yields (Br, 8%; Cl, no activity) due to the increased strength of the C–X bond when using earlier halogens, impeding the Pd insertion during oxidative addition.^47,48^ When para-substituted aryl iodide with electron-withdrawing groups (EWGs) such as trifluoromethyl or nitro was used, high yields were obtained due to the easy insertion of Pd into an electron deficient aryl-halide bond (Figure 4, products **5a** and **6**). While obtaining an amine group containing cross-coupling products often involves coupling with nitro-substituted halides followed by the reduction, in this case, even the coupling of 3-iodoaniline showed promising results (58% yield, product **7a**). In addition, direct coupling of unprotected 4-iodophenol was possible (38%, **8**), although only moderate formation of the desired product was observed. The catalytic system exhibited high activity for substrates carrying electron donating groups (EDG), resulting in high yields (80–84%, products **9** and **10a**). The choice of alkynes followed the same trend to the halide variations, with compounds containing EWG groups displaying superior activity due to the increased acidity of the terminal proton (88%, **5b**), compared to EDG-containing aryl alkynes (68%, **10b**). While the catalytic system showed good to high yields for most coupling partner combinations, substrates containing unprotected amino or hydroxy groups showed significant deviations due to unwanted side reactions.^49^ Further investigation is needed to understand how the position of the substituent at the aryl moiety affects the conversion of the starting materials. Additionally, expanding to explore a wider chemical space will provide a more comprehensive picture of potential industrial applications and deeper insights into functional group tolerances.^50^

### Erlotinib Intermediate Synthesis

**Figure 5:**
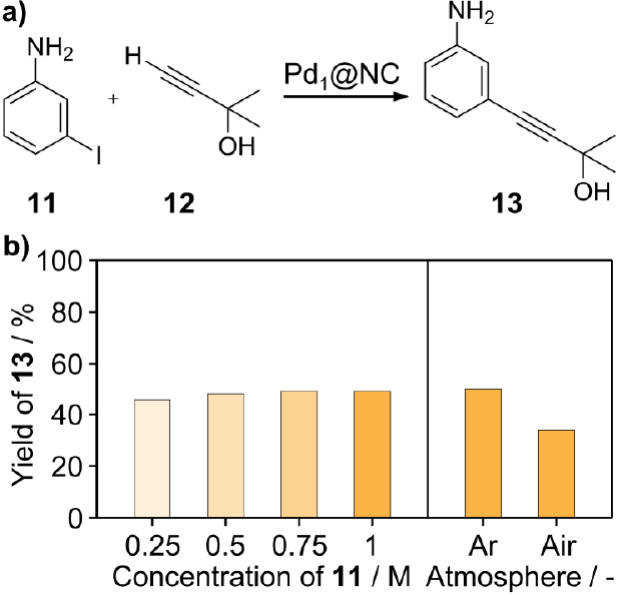
(a) Scheme of the Pd_1_@NC mediated cross-coupling of 3-iodoaniline **11** and 2-methylbut-3-yn-2-ol (**12**) toward the Erlotinib intermediate **13**. (b) Yield of alkynyl **13** in reaction media using decreasing amounts of the solvent (given as concentration of aniline **11**) and when run under protective atmosphere or in air. Standard conditions: 3-iodoaniline **11** (1 equiv), 2-methylbut-3-yn-2-ol (**12**, 1.5 equiv), NEt_3_ (2.2 equiv), MeCN (0.4 M), Pd_1_@NC (0.5 wt % Pd, 0.3 mol %), CuI (2 mol %), PPh_3_ (1 mol %) and trimethylbenzene (0.125 M) as the internal standard, at 353 K, 5 h, under Ar. Yields were determined by the GC-FID.

After optimizing the reaction environment, the Pd_1_@NC catalyst was used in the synthesis case study of the Erlotinib intermediate **13** (Figure 5a),^21^ providing a practical platform to assess its impact on the product sustainability and cost, which will subsequently inform more effective catalyst and reaction design strategies. In advance of reaction scale-up, the effect of media concentration was investigated (Figure 5b). For this, the activity of the catalytic system was monitored in reactions of decreasing solvent volumes. Here, the system exhibited a constant performance even at an aniline **11** concentration of 1 M (219 g**_11_** dm^–3^), allowing a solvent use reduction of 75% compared to common Sonogashira protocols, typically reporting a halide concentration of 0.25–0.5 M. Furthermore, we evaluated the necessity of an oxygen-free environment during the reaction. Although it is a common safety measure in industry to run reactions in the absence of oxygen to prevent the formation of explosive mixtures, we were interested in the systems’ sensitivity toward it. Two experiments were prepared using a stock solution containing all the reagents except for catalyst, CuI and PPh_3_, and the solution was then used either as-prepared or degassed using three freeze–pump–thaw cycles. The presence of oxygen in the reaction mixture led to the partial oxidation of the phosphine ligand, rendering it inactive for the promotion of the reaction. At the same time, Cu(I) can undergo oxidation, yielding Cu(II) that facilitates the homocoupling of the acetylene **12**, thereby constraining the number of possible coupling partners for the halide. Even under such conditions, the catalyst remained stable and exhibited a decrease in the final yield of just 10%, emphasizing the robustness of the system.

**Figure 6:**
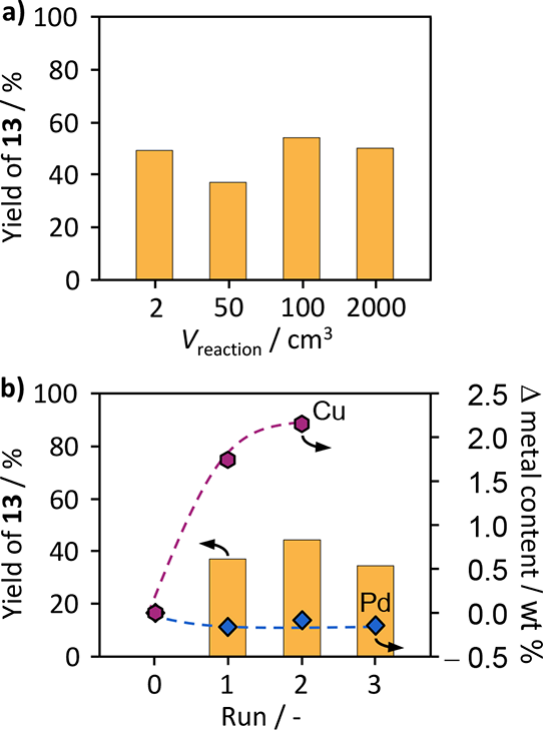
(a) Yield of alkynyl **13** obtained over Pd_1_@NC as a function of reaction volume and (b) a recycling series performed on a 50 cm^3^ scale. The changes in the palladium (blue diamond) and copper (pink hexagon) content that were determined for the recovered catalysts are shown and correspond to the axis on the right-hand side. Arrows indicate the axis applicable to the data set.

The reaction scale was stepwise increased from 2 cm^3^ (0.8 mmol**_11_**) over 50 cm^3^ and 100 cm^3^ to 2 dm^3^ (765 mmol**_11_**), with product yields of around 40% in all cases (Figure 6a). To assess the catalyst reusability under prevailing conditions, three consecutive batches were undertaken at 50 cm^3^ scale. After finishing the reaction, the catalyst was separated from the reaction mixture via filtration and washed three times with MeCN and. Subsequently, it was dried for 4 h at 373 K in air and reapplied in the Sonogashira coupling. Similar to the previous work, a slight decrease in activity can be observed, albeit not correlating with a change in the metal content (Figure 6b, dotted circles). Although a slight decrease from 0.49 to 0.36 wt % was observed after the initial use, it remains stable throughout the rest of the recycling experiments. Furthermore, the configuration of the metal atoms remained stable, showing only isolated atoms on the catalyst surface (Figure S4), ruling Pd loss and sintering out as reasons for the progressing deactivation. However, an increasing amount of copper was found to deposit on the catalyst with each use (stabilizing at about 2 wt %, Figure 6a). This was accompanied by a decrease in surface area and pore volume, determined by argon adsorption (Figure S5 and Table S14). Additionally, it was discovered during the condition optimization on the prototypical coupling toward product **3** that the catalyst experiences a postreaction weight increase of about 14% on an average after its first application. This suggests a deposition of copper species, ions like iodides and other organic compounds inside the carrier pores, which could block access to the catalyst’s active sites. Since the fouling of the catalyst is, however, potentially not an irreversible degradation of the catalyst, further tailoring of the reaction and processing conditions, e.g., ensuring (faster) conversion of depositing species or continuous flow operation, could alleviate or avoid these issues. Additionally, the activity decline could also be addressed by catalyst treatments, aiming to restore its initial state. However, few efforts have been devoted to the SAC regeneration in organic synthesis application, and deeper mechanistic understanding is necessary to engineer effective solutions.^8^

**Figure 7:**
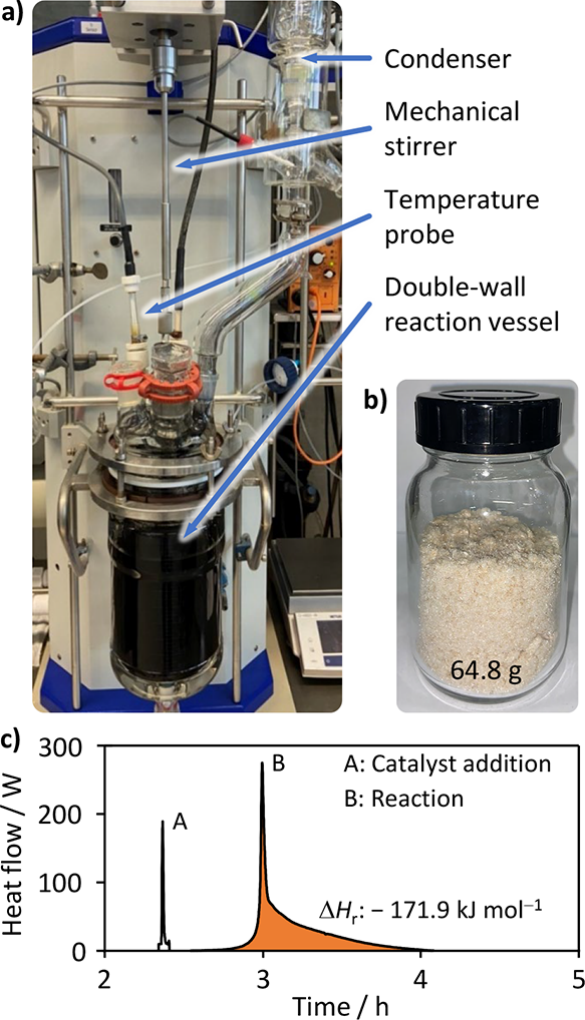
(a) Reaction calorimeter (Mettler Toledo RC-1) used for the large-scale (2 dm^3^) reaction and (b) the product 4-(3-aminophenyl)-2-methylbut-3-yn-2-ol (**13**, 64.8 g, 49%) that was obtained after recrystallization. (c) Energy profile measured with the Mettler Toledo reaction calorimeter (RC-1) during the large-scale scale reaction ran at 343 K. The first event (A, 2.4 h) takes place after the addition of the catalyst, PPh_3_ and CuI to the system. The second (B, 3.0 h) is attributed to the reaction initiation. Its area (orange) corresponds to a reaction enthalpy Δ*H*_r_ of – 171.9 kJ mol^–1^. Time measurement started (*t* = 0 h) with the beginning of heating and stirring.

The 2 dm^3^ scale coupling of 3-iodoaniline **11** and 2-methylbut-3-yn-2-ol (**12**) was performed in a RC-1 reaction calorimeter (Figure 7a), which allowed to monitor the energy release during the reaction (Figure 7c). Through prior determination of the reaction mixture heat capacity a reaction enthalpy (Δ*H*_r_) of – 171.9 kJ mol^–1^ was found.^51^ Subsequent to the separation of the catalyst by centrifugation, the product was isolated as a yellowish needlelike crystalline solid (Figure 7b, 64.8 g, 49%) by the solvent removal and recrystallization from toluene:isopropanol (10:1 *v*/*v*). Comparison of the HAADF-STEM images of the catalyst before and after the reaction showed no metal aggregation on the surface, supporting the stability of the catalyst.

While our data currently indicate a limitation to 3-iodoaniline 11 as the starting material, protocols using organometallic catalysts or carbon-supported palladium nanoparticles enable the use of the respective bromide in the synthesis of the Erlotinib intermediate **13**, with yields ranging from 75 to 92% (Table 1). In addition, some can even avoid the use of catalytic amounts of copper (I) sources. However, these studies report protocols for this reaction that involve the use of 20–30 times more palladium and require longer reaction times of 6–16 h.^18,22,52−56^ Under such conditions, it is likely that the Pd_1_@NC achieves satisfying yields using the 3-bromoaniline over the iodide, as it was demonstrated that it can perform the oxidative addition and conversion of bromides, albeit at slower rates. This is supported by the literature examples, reporting high activity of the carbon-carrier supported Pd-SACs in the Suzuki–Miyaura coupling when utilizing arylbromides.^57,58^

**Table 1:** Literature Overview of Synthesis Protocols for the Erlotinib Intermediate **13**

no.	Catalyst	Ligand, cocatalyst	Base, solvent	Conditions	X	Yield (%)	Scale (dm^3^)
1^55^	Pd(CH_3_CN)_2_Cl_2_ (2 mol %)	sSPhos (6 mol %), -	TMG, HEP:H_2_O	1 M, 353 K, 10 h	Br	92	0.05
2^22^	Pd(OAc)_2_ (3 mol %)	P(*o*-tol)_3_ (6 mol %), -	DBU, THF	1 M, 353 K, 6 h	Br	86	0.05
3^56,^^a^	10 wt % Pd/C (2 mol %)	PPh_3_ (8 mol %), CuI (4 mol %)	DME:H_2_O, K_2_CO_3_	0.3 M, 353 K, 16 h	I	78	0.1
4^68^	Pd(DPPF)Cl_2_ (2 mol %)	-	TMG, HEP	0.5 M, 333 K, 3 h	Br	99	0.05
5^52,^^b^	PdCl_2_ (0.6 mol %)	PPh_3_ (2.4 mol %), CuCl_2_ (0.2 mol %)	TMG, DMF	1 M, 348 K	Br	73	0.1
6^53^	Pd(OAc)_2_ (0.3 mol %)	PPh_3_ (3.3 mol %), CuI (0.6 mol %)	NEt_3_, -	1 M, 373 K, 7 h	Br	75	2
7^c^	Pd_1_@NC (0.3 mol %)	PPh_3_ (1.5 mol %), CuI (6 mol %)	MeCN, NEt_3_	1 M, 353 K, 3 h	I	49	2

a Stability and recyclability were not assessed.

b Reaction time not specified.

c This work.

### Environmental Footprint

As sustainability is of great concern in the modern society, assessing the environmental impact of emerging technologies is an inevitable step, particularly with regard to the chemical industry’s contribution to climate change. Here, we decided to use the recommended impact-based over mass-based metrics, as the latter, while suitable for the comparison of waste-to-product ratios, fail to capture the environmental footprint of the utilized chemicals.^59^ By this, process aspects greatly affecting the environment can be identified in early stage development, and the reaction design-induced activity differences can be linked to the product footprint. To quantify these impacts, we focus on the GWP as it is usually associated with relatively low levels of uncertainty, and climate change is considered one of the core fundamental earth system processes. The total GWP associated with the cradle-to-gate life-cycle of the process was determined using an LCA, considering contributions from the catalyst preparation, reaction, energy inputs, and waste treatment. For the calculation, it was assumed that the Pd_1_@NC can be used 10 consecutive times before it must be replaced. Even though catalyst fouling is experienced, the ICP-OES and argon adsorption of the recycled catalyst samples indicate that the fouling reaches a steady state, while no palladium leaching was observed. The slight decrease in palladium content following the initial use is assumed to originate from weakly bound palladium, which can be recovered through appropriate pretreatments of the catalyst, e.g., washing with MeCN:NEt_3_ mixtures. As such, a palladium content of 0.36 wt % is assumed for the catalyst, with the stable metal content being determined during the recycling experiments. The LCA evaluation of the system considering the data of the recycling series can be found in the Supporting Information (Table S13).

**Figure 8:**
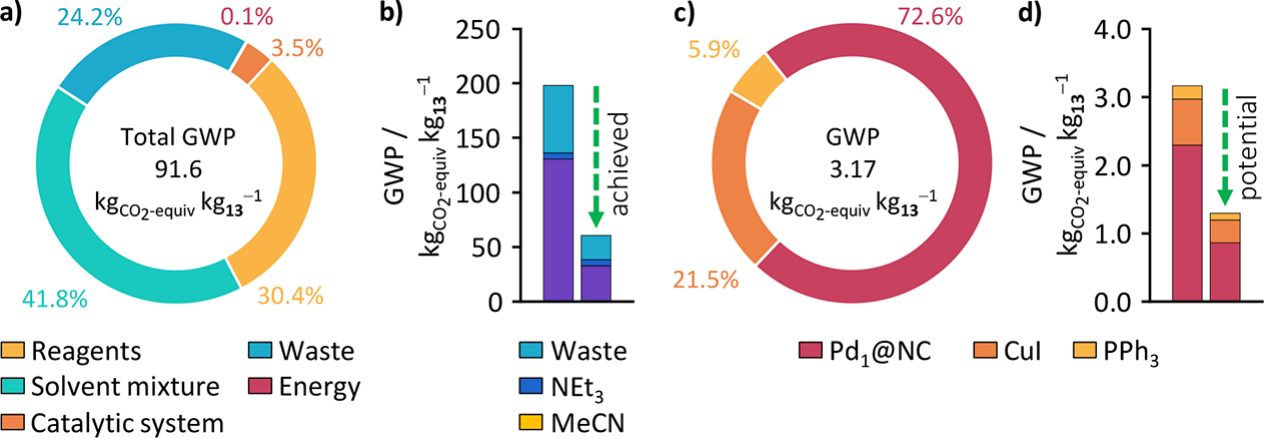
(a) Donut chart expressing the component classes’ shares of the process’ total GWP. Pd_1_@NC is assumed to be used for 10 consecutive batch reactions. Energy: Electricity and heat; Cat. System: Pd_1_@NC, CuI, and PPh_3_; Reagents: aniline **11** and alkyne **12**; Solvent mixture: MeCN and NEt_3_; Waste: Incineration of generated waste. (b) Combined GWP of solvent, base, and waste treatment before (left column) and the total CO_2_ savings enabled through the solvent reduction of 60% (green arrow, right column). (c) Donut chart expressing the GWP distribution of the Cat. System. (d) Current GWP of the Cat. System (left column) and potential savings (green arrow, right column) enabled through the reduction of CuI and PPh_3_ use and increase of the catalyst’s metal content.

With its current conditions, the solvent mixture, comprising MeCN and NEt_3_, is responsible for about 42% of the total of 91.6 kg_CO2-equiv_ kg**_13_**^–1^ (Figure 8a). Remarkably, Pd_1_@NC maintains constant performance even in more concentrated reaction media, making it possible to reduce the process solvent consumption to a quarter of common Sonogashira protocols. Like this, the product’s GWP was reduced by 137 kg_CO2-equiv_ kg**_13_**^–1^ (Figure 8b), corresponding to a total GWP reduction of 60%, and production cost savings of more than 2295 USD kg**_13_**^–1^. This stems from the fact that lowering the solvent demand not only decreases upstream contributions but also reduces the postreaction waste. Although certain environmental benefits could arise from the dedicated treatment of waste stream, e.g., recovery of scarce mineral resources like iodine,^60^ solvent waste is considered to be completely incinerated in our calculations. The possibility for solvent reduction is limited, however, by mass and heat transfer considerations. In particular, the reagents’ solubility and the reaction mixture’s heat transfer ability, which is crucial to dissipate the energy released during the reaction. The catalytic system (Pd_1_@NC, CuI and PPh_3_) with an absolute impact of 3.17 kg_CO2-equiv_ kg**_13_**^–1^ only makes up about 3.5% of the process’ total GWP.

In a breakdown of its impact (Figure 8c), it becomes apparent that about 73% is contributed by the Pd_1_@NC. This is attributed mostly to the NC, with 2.25 kg_CO2-equiv_ kg**_13_**^–1^ rather than the palladiums 0.04 kg_CO2-equiv_ kg**_13_**^–1^. Although, the palladium’s carbon footprint is about 356 times that of the carrier material (11 400 compared to 32 kg_CO2-equiv_ kg**_13_**^–1^), here, (i) it makes up less than 0.5 wt % of the catalyst, and more importantly (ii) 98% are recovered and the catalyst’s end of usable lifetime. Accordingly, only 2% of the catalyst’s initial palladium content must be replaced to resynthesize the fresh catalyst, leading to a diminishing contribution of the palladium that decreases even further with every time the catalyst can be reused. This is important to understand as it also reveals an opportunity to reduce the already small carbon footprint and cost of the catalyst even further, namely, by increasing the palladium content. Assuming the consistent activity when increasing the Pd_1_@NC, the necessary mass of catalyst and as such the use of carrier material would decrease proportionally. In fact, metal contents of up to 20 wt % have already been reported for different metal–carrier combinatioons.^61^ The palladium source used to impregnate the catalyst surface has an impact as well, even though it is mostly governed by the impact of the palladium itself rather than further chemical modifications (Table S11). PdCl_2_ and Pd(NO_3_)_2_ present the best choices with 1213.34 and 1213.48 kg_CO2-equiv_ mol^–1^, a result of them being used as the starting material for most other potential precursor. To offer the system consistency and optimal comparability to other protocols, the here presented economic and environmental impact approximations were using the cost and GWP of elemental palladium.

Another interesting insight that is obtained here is the small ecological (0.87 kg_CO2-equiv_ kg**_13_**^–1^) and economic (28 USD kg**_13_**^–1^, Table S13) effect CuI and PPh_3_ have on the API. This puts the trend within the Sonogashira coupling community, seeking copper- and ligand-free systems, into a new perspective as their environmental impact is insignificant, at least in terms of GWP. It is also important to realize that the amounts of CuI and PPh_3_ that were used for the large-scale reaction were not optimized for process efficiency but deliberately chosen higher than necessary. This was to avoid any effects on performance stemming from variations in the Pd:CuI:PPh_3_ ratios. As seen in the study of CuI:Pd and PPh_3_:Pd ratios (Figure 3a,b), a reduction of 50% should be possible for both without negatively impacting activity. This, in combination with a small increase of the catalyst’s palladium content to 1 wt %, would entail GWP savings for the catalytic system of almost 60% with the potential for even greater reductions (Figure 8d).

Estimating the required time for implementation of SACs in large-scale C–C coupling applications is a complex task due to various external factors, including (i) the similarity to existing technologies, (ii) the associated infrastructural demands and their costs, (iii) the impact of regulations on the development and final process, and (iv) the financial value of overhauling the process in question. In the best-case scenario, the catalyst can simply be exchanged with only minor adaptations to the manufacturing infrastructure, provided it works optimally under the previously employed conditions. When switching from homogeneous to heterogeneous catalysis, an infrastructural modification could be the installation of suitable filtration units. The time to implement a novel catalytic material would then be mostly dependent on the time needed for its commercialization.

## Conclusions

In summary, this work investigated the influence of reaction environments on the performance of a single-atom heterogeneous catalyst in organic synthesis, providing valuable insights for maximizing their catalytic potential and enabling efficient metal recovery in these applications. The study of different solvents revealed notable variations in performance, distinct from the previous reports on homogeneous catalysts. Interestingly, attempts to correlate activity with the solvent’s Kamlett–Taft parameter proved unsuccessful, underscoring the complex interplay among the solvent, reagents, and active center of a heterogeneous catalyst. Furthermore, inorganic bases such as carbonates and acetates displayed comparable yields to secondary and tertiary amines, presenting noncorrosive alternatives for large-scale applications.^62^ The catalytic system also exhibited successful coupling reactions involving electron-withdrawing and -donating groups containing halides and acetylenes. Further, the system optimization will entail the evaluation of utilizing continuous flow operation and suitable catalyst recovery, as well as regeneration methods. To verify the practical potential of the catalyst, we demonstrated the scalability of its production without any structural deviations from small-scale preparations and stable performance in a multigram synthesis of an API intermediate. A life-cycle assessment revealed the low environmental impact of Pd_1_@NC and identified areas for further savings, such as additive or solvent reduction. Further, we found that the production cost and GWP of intermediate **13** are hardly affected by the use of PPh_3_ or CuI. This work clearly demonstrates that although the research centered around the application of SACs for fine-chemical synthesis is still in an early stage, these materials present significant opportunities for sustainable chemistry. We envision that our catalyst can be complemented by other advances in sustainable organic transformations, such as water-based chemistry utilizing micelle forming surfactants.^63−66^

## Supporting Information Available

Experimental procedures for the catalyst preparation, evaluation in the Sonogashira coupling, and the LCA procedure, supporting catalyst characterization, performance, and analytical data. (PDF)
