# Supplementary material for: Reaction Environment Design for Multigram Synthesis via Sonogashira Coupling over Heterogeneous Palladium Single-Atom Catalysts
Source: ACS Sustain Chem Eng. 2023 Nov 22;11(48):16935–45. doi: 10.1021/acssuschemeng.3c04183 (PMC10698743; doi:10.1021/acssuschemeng.3c04183)
Supplement: Supplementary file 1 — sc3c04183_si_001.pdf [file sc3c04183_si_001.pdf]

Supporting Information

Reaction Environment Design for Multigram Synthesis via Sonogashira Coupling over Heterogeneous Palladium Single-Atom Catalysts

Dario Poier[a], Dario Faust Akl[b], Elysia Lucas[b], Alicia Rodrigues Machado[a], Georgios Giannakakis[b], Sharon Mitchell[b], Gonzalo Guillén-Gosálbez[b], Roger Marti*[a], Javier Pérez-Ramírez*[b]*

[a] Institute of Chemical Technology, Haute école d'ingénierie et d'architecture Fribourg, HES-SO University of Applied Sciences and Arts Western Switzerland, 1700 Fribourg, Switzerland.

[b] Institute for Chemical and Bioengineering, Department of Chemistry and Applied Biosciences, ETH Zurich, Vladimir-Prelog-Weg 1, 8093 Zurich, Switzerland.

* Corresponding authors

Sharon Mitchell, E-mail: sharon.mitchell@chem.ethz.ch

Roger Marti, E-mail: roger.marti@hefr.ch

Javier Pérez-Ramírez, E-mail: jpr@chem.ethz.ch

Total number of pages: 37

Total number of figures: 15

Total number of tables: 14

Table of Contents

Abbreviations	S3
Catalyst Synthesis	S4
Cross-Coupling Protocols	S5
LCA	S6
Solvent and Base Screening Results	S8
Source Materials and Assessment of the Large-Scale Synthesis	S10
Sonogashira Coupling Mechanism and Erlotinib Synthesis	S15
Synthesis Paths of the Compounds for Large-Scale Synthesis	S16
Additional Catalyst Characterization and Catalytic Data	S17
LCA Upstream Analysis and Uncertainties	S23
NMR Data	S25
NMR Spectra	S29
References	S36

Abbreviations

**General**	
API	Active pharmaceutical ingredient
CAS	Chemical abstracts service
GWP	Global warming potential
IPCC	Intergovernmental panel on climate change
LCA	Life-cycle assessment
LCIA	Life-cycle impact assessment
SAC	Single-atom catalyst
**Techniques**	
AC-ADF	Aberration corrected annular dark-field
EDX	Energy dispersive X-ray spectroscopy
GC-FID	Gas chromatography flame ionization detection
HAADF	High-angle annular dark-field detection
ICP-OES	Inductively coupled plasma optical emission spectroscopy
NMR	Nuclear magnetic resonance spectroscopy
STEM	Scanning transmission electron microscopy
TGA	Thermogravimetric analysis
**Compounds**	
AC	Activated carbon
ClPh	Chlorobenzene
DCE	1,2-dichloroethane
DI water	Deionized water
DMF	Dimethylformamide
DIPEA	Diisopropylethylamine
EtOAc	Ethyl acetate
EtOH	Ethanol
H_2NBu; $HNBu_2$; NBu_3	*n*-butylamine; di-*n*-butylamine; tri-*n*-butylamine
KOAc	Potassium acetate
KOMe	Potassium methanolate
MeCN	Acetonitrile
MePh	Toluene
NC	Nitrogen-doped carbon
NEt_3	Triethylamine
PG	Propylene glycol / Propane-1,2-diol
PPh_3	Triphenylphosphine

Catalyst Synthesis

Nitric acid (> 65 wt%, puriss.) and dicyandiamide (99%) were purchased from Sigma-Aldrich, activated carbon (AC, Norit Rox 0.8) from Cabot Corporation, palladium (99.998%), $(NH_3)_4Pd(NO_3)_2$ (5 wt% water), $Pd(NO_3)_2 \cdot 2H_2O$ (41 wt% Pd) from abcr. The reagents for the cross-coupling reactions were purchased from Chemie Brunschwig AG. All chemicals were used without further purification.

Preparation of Nitrogen-Doped Carbon (NC) Carrier.[1] As the first step of nitrogen incorporation, AC (23.0 g) was sieved (sieve fraction < 0.2 mm) and refluxed in nitric acid (4 M, 0.46 dm^3) at 353 K for 16 h. The mixture was poured into DI water (273 K, 0.50 dm^3), filtered, washed copiously with DI water (4.6 dm^3), and dried overnight (338 K). The acid-activated carbon was added to a solution of dicyandiamide (69.0 g, 0.82 mol) in acetone (0.69 dm^3), which was subsequently evaporated at 353 K under constant stirring. Finally, the dried solid was gently crushed, transferred to ceramic boats, and carbonized in flowing nitrogen (723 K, 3 h hold, then 923 K, all ramps 5 K min^{-1}) to obtain NC (55.2 g) as a black powder.

Preparation of Pd_1@NC Single-Atom Catalyst (SAC). $(NH_3)_4Pd(NO_3)_2$ (5.00 mg, 0.02 mmol) and DI water (15 cm^3) were added to a sonicated (30 min) suspension of as-prepared NC (1 g) in DI water (20 cm^3) and vigorously stirred overnight. The suspension was then subjected to repeated cycles (20) of microwave irradiation (100 W for 15 s) with pressurized air cooling (45 s between cycles), maintaining a sample temperature of 30 °C. Afterward, the solid was separated through centrifugation, washed with water (5×5 cm^3) and ethanol (5×5 cm^3), dried overnight (353 K), and annealed in a tubular oven under nitrogen flow (573 K, 5 h hold, 5 K min^{-1} ramp) to obtain the Pd_1@NC (1 g) as a black solid.

For the preparation of larger amounts of Pd_1@NC, the protocol was adapted as follows. $Pd(NO_3)_2$ (0.25 g, 1.08 mmol) and DI water (0.2 cm^3) were added to a sonicated (30 min) suspension of as-prepared NC (50 g) in DI water (0.6 dm^3). The volume of the suspension was adjusted to 1 dm^3 with DI water and stirred overnight. After filtration, the solids were washed with DI water (6 dm^3) and dried at 383 K. Finally, the solid was annealed in a static nitrogen atmosphere (573 K, 5 h, 5 K min^{-1} ramp) to obtain the Pd_1@NC (50 g) as a black solid.

Cross-Coupling Protocols

1,3,5-trimethylbenzene was used as an internal standard for the gas chromatography flame ionization detection (GC-FID) analysis of the reaction solution.

Pd_1@NC Recycling Experiments. 3-Iodoaniline (compound **11** in Figure 5, 3.67 g, 16.7 mmol), 2-methylbut-3-yn-2-ol (**12**, 1.55 g, 18.5 mmol), triethylamine (NEt_3, 4.23 g, 41.9 mmol), 1,3,5-trimethylbenzene (0.25 g, 2.10 mmol), and acetonitrile (MeCN, 13.7 g, 17.4 cm^3, 0.96 M) were loaded into a 100 cm^3 round-bottom flask. Pd_1@NC (0.5 wt% Pd, 1.02 g, 0.05 $mmol_{Pd}$), copper(I) iodide (CuI, 191 mg, 1.00 mmol), and triphenylphosphine (PPh_3, 66.0 mg, 0.25 mmol) were added to the solution and vigorously stirred for 5 h at 353 K under a protective atmosphere (Ar). After cooling to room temperature, the Pd_1@NC was separated from the reaction mixture by centrifugation, washed with MeCN (3×15 cm^3) and ethyl acetate (EtOAc, 3×15 cm^3), and dried in vacuo at 373 K for 2 h. The recovered Pd_1@NC was then reused, following the same procedure as described above. 4-(3-aminophenyl)-2-methylbut-3-yn-2-ol (**13**) was determined in the reaction solution by GC-FID analysis, corresponding to a yield of 37% (first use), 44% (second use) and 34% (third use).

Large-Scale Synthesis of Erlotinib Intermediate. 3-Iodoaniline (**11**, 167 g, 765 mmol), 2-methylbut-3-yn-2-ol (**12**, 96.4 g, 1.15 mol), NEt_3 (193 g, 1.91 mol), 1,3,5-trimethylbenzene (11.5 g, 95.6 mmol), and MeCN (700 g, 891 cm^3) were loaded into a Mettler Toledo RC-1 reaction calorimeter. Pd_1@NC (0.5 wt% Pd, 45.8 g, 2.15 $mmol_{Pd}$), CuI (8.79 g, 46.2 mmol), and PPh_3 (2.90 g, 11.1 mmol) were added to the solution, and vigorously stirred at 343 K. Reaction progress was monitored by GC-FID. Reaching full conversion of **11** after 3 h, the reaction mixture was cooled to room temperature, the Pd_1@NC separated from the reaction mixture by centrifugation, washed with MeCN (3×200 cm^3) and EtOAc (3×200 cm^3), dried in vacuo at 373 K for 2 h and analyzed by inductively coupled plasma optical emission spectroscopy (ICP-OES) and high-angle annular dark-field detection scanning transmission electron microscopy (HAADF-STEM). After taking an aliquot for GC-FID analysis, volatile components were removed from the reaction solution under reduced pressure, the residue treated with demineralized H_2O (500 cm^3) and the aqueous phase extracted with EtOAc (3×500 cm^3). The combined organic phases were dried over Na_2SO_4, the solvent removed under reduced pressure and the brown residue subjected to recrystallization from toluene:isopropanol (10:1 *v*/*v*) to yield 4-(3-aminophenyl)-2-methylbut-3-yn-2-ol (**13**, 64.8 g, 370 mmol, 49%) as yellowish crystalline solid.

Life-Cycle Assessment (LCA) Protocol

Framework and Methodology. The LCA conducted in this work followed the principles and framework defined in the ISO 14040:2006 standards.[2] The ISO 14040:2006 defines the following four phases for LCA: (i) definition of goal and scope, (ii) inventory analysis, (iii) impact assessment, and (iv) interpretation. The goal of the analysis was to investigate the relative contributions of process inputs and outputs to the global warming potential (GWP) of the large-scale synthesis of **13**. The functional unit was defined as 1 kg of **13** with a cradle-to-gate system boundary. This system boundary includes synthesis of **13** in a 100-liter batch reactor, isolation of intermediate **13**, waste treatment of spent solvent mixture, palladium recovery and refining, waste treatment of unrecovered palladium and catalyst resynthesis.

In the second phase of the LCA, an inventory of material and energy flows in and out of the defined system was undertaken. Material inputs included all reagents, solvent, base, and catalysts required to synthesize Pd_1@NC and intermediate **13**. Energy requirements comprised of reaction heating, as well as electricity for reactor tank stirring and separation of the heterogeneous catalyst by filtration. Lastly, the treatment of major waste streams was considered – namely, the spent solvent mixture waste stream (via incineration) and the lost palladium (assumed to reach landfill) due to incomplete recovery at the metal refining and recovery stage prior to catalyst resynthesis. The amounts of material inputs into the system were obtained from experimental data and energy requirements were estimated using approaches from literature.[3] Existing unit processes in the life cycle inventory database ecoinvent v3.9[4,5] were used to represent all material and energy inputs, as well as the waste treatment activities.

The GWP, expressed in terms of $kg_{CO2\text{-}eq.}$, of producing 1 kg of **13** was estimated using the Intergovernmental Panel on Climate Change (IPCC) 2013 GWP100 (over a 100-year time horizon) life-cycle impact assessment (LCIA) method. The GWP100 method characterizes and quantifies all greenhouse gas emissions associated with the production system – encompassing emissions embedded in all material and energy inputs, as well as emissions associated with waste treatment. LCIA methods convert environmental exchanges (e.g., carbon dioxide or methane emissions to air) to environmental impacts (e.g., global warming) using characterization factors. All impact calculations were undertaken using Brightway[6], an open-source software package for LCA and environmental impact assessment.

GWP Uncertainties. It is important to acknowledge the uncertainty of the GWP impact estimate presented in this work. One of the main contributors of quantitative uncertainty of LCA results is the potential variability of inventory flows. Inventory flows refer to material and energy requirements from other production processes, as well as their corresponding emissions to the environment. The uncertainty of life cycle inventories therefore includes uncertainty associated with foreground data (direct inputs and outputs of the system under study) and with the background systems they are linked to.

In this analysis, we do not consider uncertainty in the foreground data as knowledge on the probability distributions of each foreground system input (i.e., the potential distribution of the amounts of material and energy inputs for the large-scale production of **13**) is currently very limited at this current stage of laboratory scale development. The uncertainty range on the GWP result we quantify captures only the uncertainties from the background system processes in ecoinvent v3.9[4,5]. Background uncertainties were propagated using Monte Carlo analysis in Brightway (1,000 simulation runs), giving a range of 85.8 to 164.1 $kg_{CO2\text{-}eq.}\ kg_{\mathbf{13}}^{-1}$ (Figure S7). The Monte Carlo analysis was carried out in Brightway, involving 1000 simulations. Although the conclusions regarding the topmost contributors to the total impact remain unchanged, it must be appreciated that the GWP impact of 13 could potentially deviate by −9 to +105% to the nominal deterministic case (91.7 $kg_{CO2\text{-}eq.}\ kg_{\mathbf{13}}^{-1}$).

Future work on more detailed assessments of the synthesis system of **13**, and the environmental implications of any further optimization, could include a more comprehensive sensitivity analysis on possible input amount ranges of catalyst, reagents, chemical environment components and energy when there is increased knowledge on their potential distribution when the process is scaled up.

Solvent and Base Screening Results

Table S1. Effect of varying the solvent on the yield of alkynyl **3**.[7,8]

**Solvent**[a]	α□	β	π*	**Yield**[b] **/ %**
MeCN	0.19	0.31	0.75	94
EtOH	0.83	0.75	0.51	65
$OP(OEt)_3$	0.00	0.77	0.72	12
$OP(OEt)_3$:PG (2:1)	0.28	0.77	0.73	26
$OP(OEt)_3$:PG (1:2)	0.55	0.78	0.75	36
PG	0.83	0.78	0.76	57
DCE	0.00	0.00	0.81	14
DCE:DMF (2:1)	0.00	0.23	0.83	6
DCE:DMF (1:2)	0.00	0.46	0.86	3
DMF	0.00	0.69	0.88	16
MePh	0.00	0.11	0.54	55
MePh:ClPh (2:1)	0.00	0.10	0.60	63
MePh:ClPh (1:2)	0.00	0.08	0.65	63
ClPh	0.00	0.07	0.71	53

[a] The solvents (except for MeCN and EtOH) were chosen as the $OP(OEt)_3$:PG, DMF:DCE and MePh:ClPh solvent pair that mostly differ in their proticity α, basicity β and polarizability/polarity π* (all ranging from 0 to 1), respectively. The solvent mixtures (neat solvent, 2:1, 1:2) are arranged in order of ascending parameter value.

[b] Reaction conditions: iodobenzene (**1**, 1 eq.), phenylacetylene (**2**, 1.5 eq.), NEt_3 (2.2 eq.), solvent (0.4 M), Pd_1@NC (0.5 wt% Pd, 0.2 mol%), CuI (2 mol%), PPh_3 (1 mol%) and trimethylbenzene (0.125 M) as internal standard, at 353 K, 24 h, under Ar. Yields were determined by GC-FID.

Table S2. Effect of varying the base on the yield of alkynyl **3**.

Base	Yield[a] / %
NEt_3	94
H_2NBu	21
$HNBu_2$	65
NBu_3	68
DIPEA	75
Na_2CO_3	26
K_2CO_3	97
Cs_2CO_3	92
KOAc	80
KOMe	2
KOH	90

[a] Reaction conditions: iodobenzene (**1**,1 eq.), phenylacetylene **2** (1.5 eq.), base (2.2 eq.), MeCN (0.4 M), Pd_1@NC (0.5 wt% Pd, 0.2 mol%), CuI (2 mol%), PPh_3 (1 mol%) and trimethylbenzene (0.125 M) as internal standard, at 353 K, 24 h, under Ar. Yields were determined by GC-FID.

Source Materials and Assessment of the Large-Scale Synthesis

Table S3. Source materials assumed in the LCA to produce 1 kg of Pd_1@NC. Based on the synthesis protocol on page S3, assuming a palladium content of 0.36 wt%.

**Compound**	**CAS**	**Mass / kg**
Pd	7440-05-3	0.0036
AC	7440-50-8	0.953
HNO_3	7553-56-2	2.961
H_2O	7732-18-5	0.510
CO_2	124-38-9	1.247
N_2	7727-37-9	0.208
CaO	1305-78-8	0.833

Table S4. Source materials assumed in the LCA to produce 1 kg of CuI. Based on the synthesis path shown in Scheme S3a.

**Compound**	**CAS**	**Mass / kg**
Cu	7440-50-8	0.333
I_2	7553-56-2	0.666

Table S5. Source materials assumed in the LCA to produce 1 kg of PPh_3. Based on the synthesis path shown in Scheme S3b.

**Compound**	**CAS**	**Mass / kg**
Na	7440-23-5	0.524
Cl_2	7782-50-5	1.216
C_6H_6	71-43-2	0.893
P_4	12185-10-3	0.118

Table S6. Source materials assumed in the LCA to produce 1 kg of MeCN. It is obtained as a side-product during the production of acrylonitrile through the Sohio process.

Compound	CAS	Mass / kg
O_2	7782-44-7	111.4
C_3H_6	115-07-1	97.63
NH_3	7664-41-7	39.51

Table S7. Source materials assumed in the LCA to produce 1 kg of NEt_3. Based on the synthesis path shown in Scheme S3c.

Compound	CAS	Mass / kg
C_2H_6O	64-17-5	1.365
NH_3	7664-41-7	0.168

Table S8. Source materials assumed in the LCA to produce 1 kg of 2-methylbut-3-yn-2-ol (**12**). Based on the synthesis path shown in Scheme S3d.

Compound	CAS	Mass / kg
$OC(CH_3)_2$	67-64-1	0.691
C_2H_2	74-86-2	0.310
KOH	1310-58-3	0.007

Table S9. Source materials assumed in the LCA to produce 1 kg of 3-iodoaniline **11**. Based on the synthesis path shown in Scheme S3e.

Compound	CAS	Mass / kg
H_2	12385-13-6	0.027
I_2	7553-56-2	1.083
S_8	7704-34-9	0.183
SO_2	7446-09-5	0.305
Na_2CO_3	497-19-8	0.504
K_2CO_3	584-08-7	1.315
H_2SO_4	7664-93-9	0.466
HNO_3	7697-37-2	0.450
C_6H_6	71-43-2	0.371

Table S10. Cost and GWP breakdown for the reagents of the Pd_1@NC synthesis.

**Contributing Item**	**Cost[a] / USD $kg_{cat.}^{-1}$**	**GWP / $kg_{CO2\text{-}eq.}$ $kg_{cat.}^{-1}$**
AC	17.30	3.034
HNO_3	34.52	3.013
Dicyandiamide	45.51	14.27
Pd	144.5	41.04
Energy	12.95	21.53
Total	254.8	82.89

[a] Based on current prices at Brunschwig Chemicals and a palladium value of 40.14 USD g_{Pd}^{-1} (September 2023).

Table S11. GWP of different palladium sources for the preparation of the Pd_1@NC. Based on the synthesis paths shown in Scheme S4.

**Compound**	**CAS**	**GWP / $kg_{CO2\text{-}eq.}$ kg^{-1}**	**GWP / $kg_{CO2\text{-}eq.}$ mol^{-1}**
Pd	7440-05-3	11401.03	1213.30
$PdCl_2$	7647-10-1	6842.29	1213.34
$(NH_3)_4PdCl_2$	13933-31-8	4974.53	1213.54
$Pd(NO_3)_2$	10102-05-3	5280.57	1213.48
$(NH_3)_4Pd(NO_3)_2$	13601-08-6	4074.08	1213.67
$Pd(O_2CCH_3)_2$	3375-31-3	5420.20	1213.75
$PdSO_4$	13566-03-5	6013.32	1213.49

Table S12. Cost and GWP of the Pd_1@NC (0.36 wt% Pd) preparation in relation to the produced Erlotinib intermediate **13**.

Scenario	Cost[a, b] / USD kg_{13}^{-1}	GWP[b] / $kg_{CO2\text{-}eq.}$ kg_{13}^{-1}	Cost[a, c] / USD kg_{13}^{-1}	GWP[c] / $kg_{CO2\text{-}eq.}$ kg_{13}^{-1}
1 use	61.21	23.09	137.8	44.85
3 uses	20.40	7.695	45.95	14.95
3 uses, deactivation	27.58	10.40	62.09	20.20
10 uses	6.122	2.309	13.78	4.485

[a] Based on current prices at Brunschwig Chemicals and a palladium value of 40.14 USD g_{Pd}^{-1} (September 2023).

[b] Pd_1@NC is assumed to be stable towards leaching; 98% of the palladium is recovered after the catalyst's end of usable lifetime and used for resynthesizing the catalyst; recovery procedure is omitted.

[c] Pd_1@NC is assumed to be stable towards leaching; palladium is not recovered at the end of the catalyst's usable lifetime.

Table S13. Costs of reagents and GWP of resources for the synthesis of Erlotinib intermediate **13**.

**Contributing Item**	**Cost[a] / USD kg_{13}^{-1}**	**Cost Share / %**	**GWP / $kg_{CO2\text{-}eq.}$ kg_{13}^{-1}**	**GWP Share / %**
Pd_1@NC	27.58[b]	0.8	10.40	10.4
CuI	23.46	0.7	0.681	0.7
PPh_3	5.576	0.2	0.186	0.2
MeCN	765.6	23.4	32.55	32.6
NEt_3	76.19	2.3	5.786	5.8
Aniline **11**	2364	72.2	25.53	25.6
Acetylene **12**	8.183	0.3	2.352	2.4
Waste	0.414	< 0.1	22.18	22.2
Energy	0.032	< 0.1	0.081	< 0.1
Total	3270	100.0	99.75	100

[a] Based on current prices at Brunschwig Chemicals and a palladium value of 40.14 USD g_{Pd}^{-1} (September 2023).

[b] Considering Pd_1@NC stable towards leaching, an initial palladium content of 0.36 wt%, 3 uses with a linear activity decline of 22.8% each time and 0.98% metal recovery at the end of the catalyst's usable lifetime.

Sonogashira Coupling Mechanism and Erlotinib Synthesis

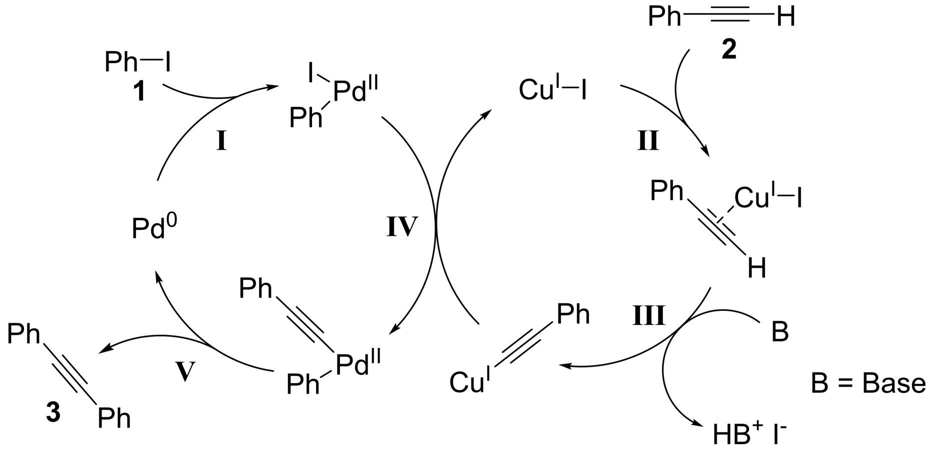

Scheme S1. Broadly accepted mechanism for the palladium-copper co-catalyzed Sonogashira coupling. A palladium center in the Pd^{0} oxidation state (either in the as-prepared catalyst or generated *in situ*) inserts into the aryl–halide bond during the oxidative addition (**I**), while the copper iodide activates the carbon–carbon triple bond for deprotonation through the base (**II**), generating a copper acetylide (**III**). Subsequently, the Pd^{II} and copper acetylide undergo transmetalation (**IV**) followed by a reductive elimination of the final product (**V**).

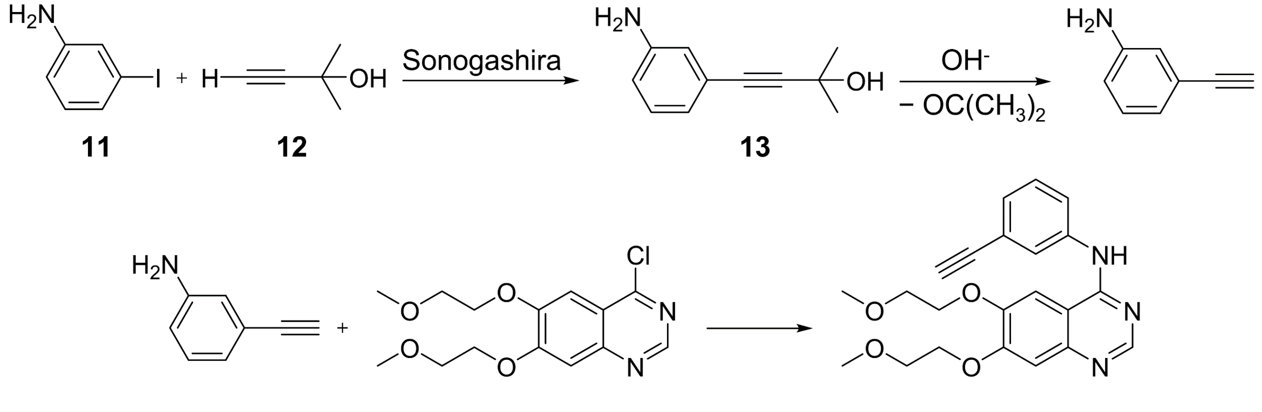

Scheme S2. Synthesis scheme for the preparation of Erlotinib via intermediate **13**, whose preparation is reported in this work. Subsequent steps consist of the triple bond deprotection in basic conditions, followed by the C–N coupling.[9]

Synthesis Paths of the Compounds for Large-Scale Synthesis

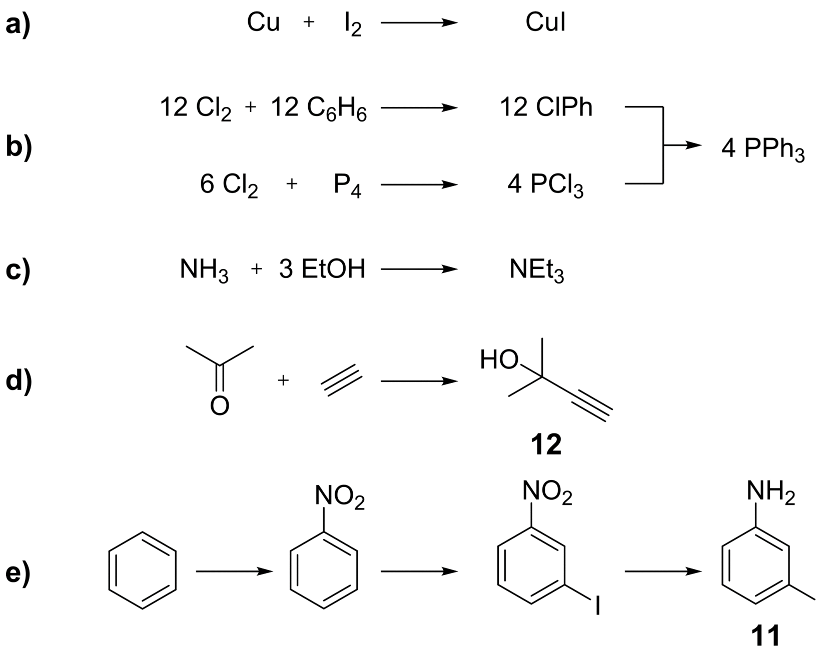

Scheme S3. Synthesis paths for **a)** CuI, **b)** PPh_3, **c)** NEt_3, **d)** 2-methylbut-3-yn-2-ol (**12**) and **e)** 3-iodoaniline **11** that were considered for the GWP calculations.

a) $Pd + Cl_2 \longrightarrow PdCl_2$

b) $PdCl_2 + 4\ NH_3 \longrightarrow (NH_3)_4PdCl_2$

c) $Pd + {}^{8}/_{3}\ HNO_3 \longrightarrow Pd(NO_3)_2$

d) $Pd(NO_3)_2 + 4\ NH_3 \longrightarrow (NH_3)_4Pd(NO_3)_2$

e) $Pd(NO_3)_2 + 2\ H_3COOH \longrightarrow Pd(OAc)_2$

f) $Pd(NO_3)_2 + H_2SO_4 \longrightarrow PdSO_4$

Scheme S4. Synthesis paths for **a)** $PdCl_2$, **b)** $(NH_4)_2PdCl_2$, **c)** $Pd(NO_3)_2$, **d)** $(NH_4)_2Pd(NO_3)_2$ and **e)** $Pd(OAc)_2$ and **f)** $PdSO_4$ that were considered for the GWP calculations.

Additional Characterization and Catalytic Data

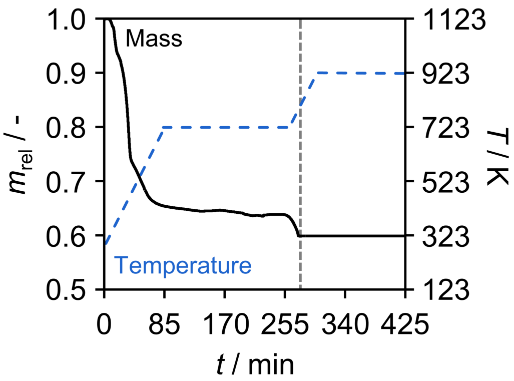

Figure S1. Thermogravimetric analysis (TGA) of the AC:dicyandiamide mix during the NC yielding carbonization. The analysis shows that amount of NC obtained after the treatment is approximately 60 wt% of the initial AC:dicyandiamide mix. It becomes apparent that the 2 h period at 923 K can be avoided, as the sample mass loss ceases during the second temperature ramp already. Temperature settings were as follows: from 298 K to 723 K with 5 K min^{-1}, 3 h hold at 723 K, with 5 K min^{-1} to 923 K and 2 h hold at 923 K, before cooling down to room temperature again.

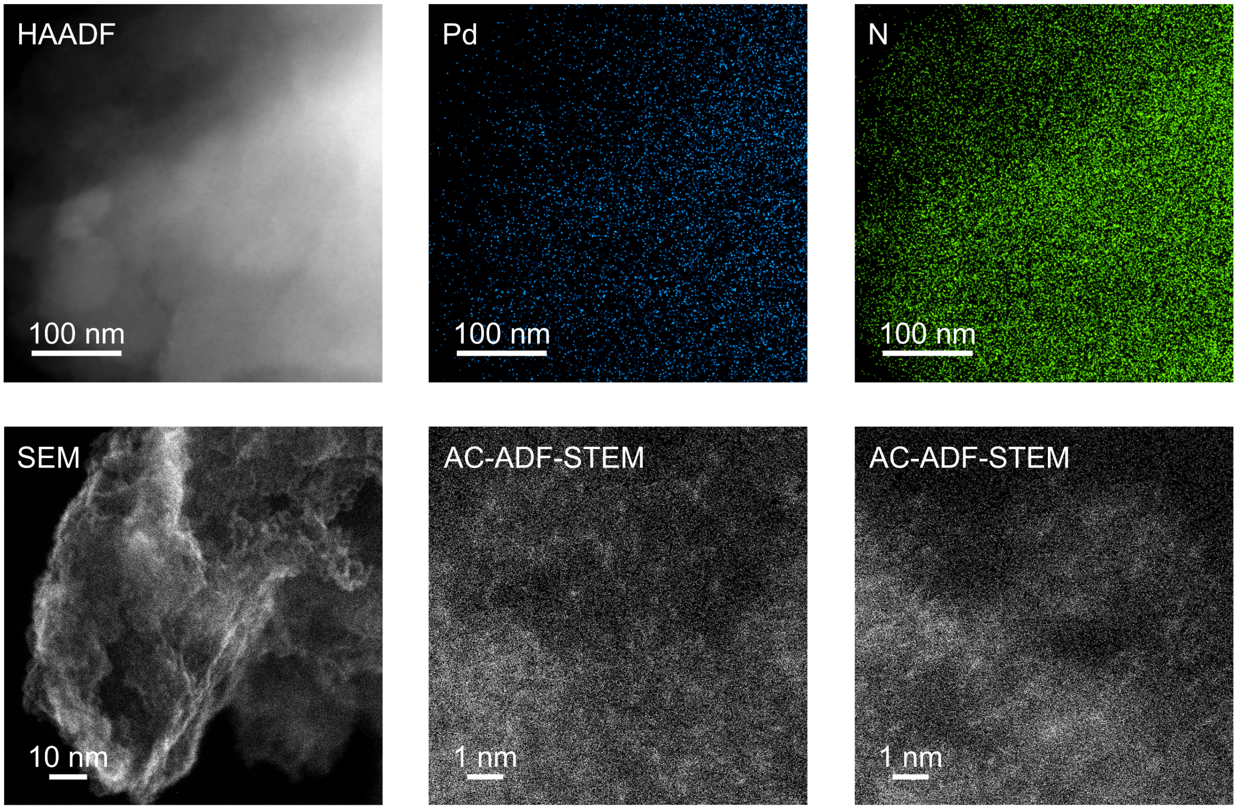

Figure S2. HAADF-STEM image and EDX maps (top row), SEM, and AC-ADF-STEM images (bottom row) of the as-prepared Pd_1@NC show a uniform distribution of palladium with no visible nanoparticles.

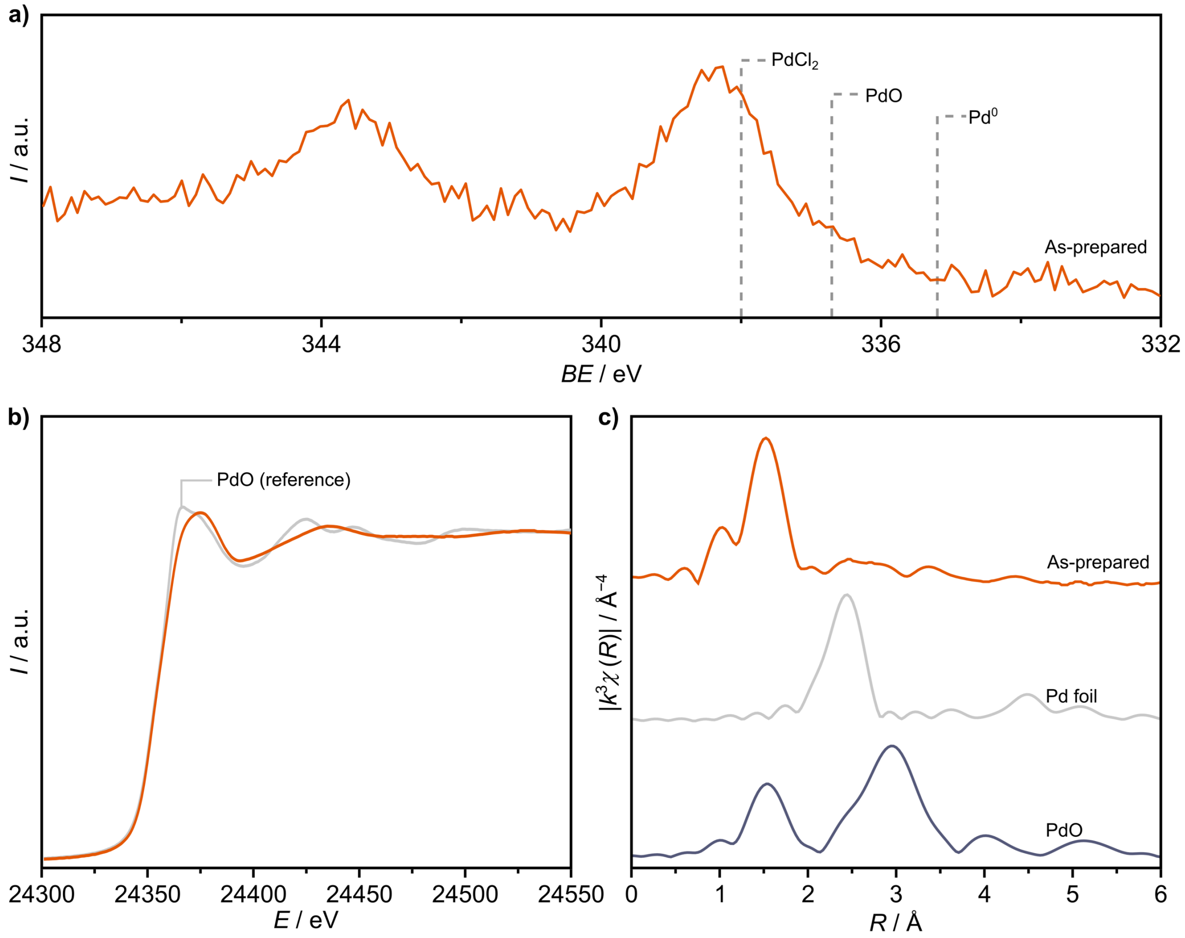

Figure S3. a) Pd 3*d* X-ray photoemission spectrum (XPS), b) X-ray absorption near-edge structure (XANES), and c) Fourier-transformed extended X-ray absorption fine structure (EXAFS) of the as-prepared Pd_1@NC catalyst (references shown overlaid, where relevant). As corroborated by XPS and XANES, Pd adopts a highly oxidized electronic state when supported on NC. Inspection of the EXAFS spectrum reveals Pd–O scattering and corroborates the absence of contributions stemming from Pd–Pd.

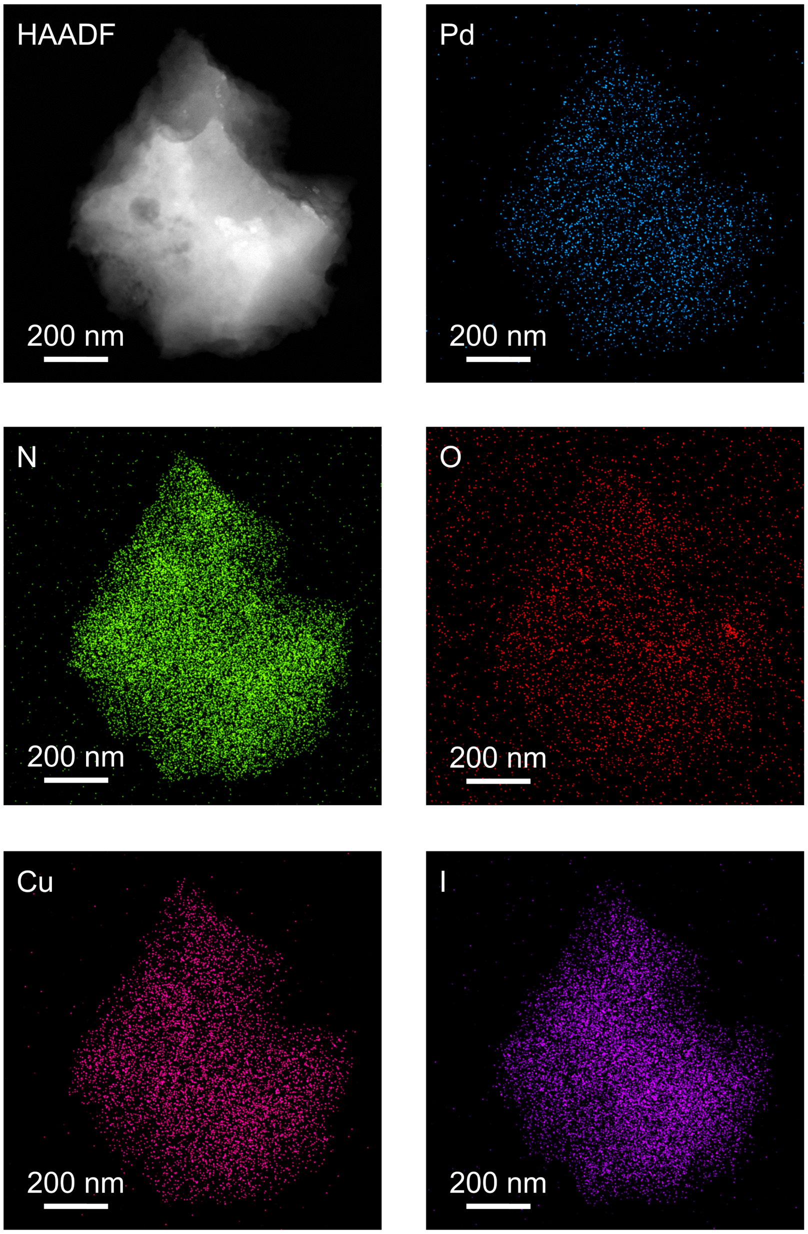

Figure S4. HAADF-STEM image and EDX maps of the Pd_1@NC after the third consecutive use in the synthesis towards product **13**. No nanoparticles are visible and palladium as well as copper species are observed to be uniformly distributed.

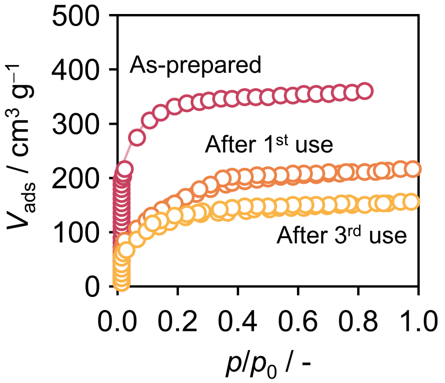

Figure S5. Argon sorption isotherms at 77 K of the catalyst samples at different stages during the recycling series. The decreased surface area and pore volume observed likely originates from the deposition of organic compounds and copper species inside the carrier pores, as no catalyst degradation was experienced.

Table S14. Ar sorption results for the as-prepared catalyst and during the 50 cm^3 recycling series obtained samples.

**Pd_1@NC sample**	**S_{BET} / m^2 g^{-1}**	**V_{pore} / cm^3 g^{-1}**
As-prepared	1025	0.33
After 1st use	521	0.07
After 3rd use	408	0.11

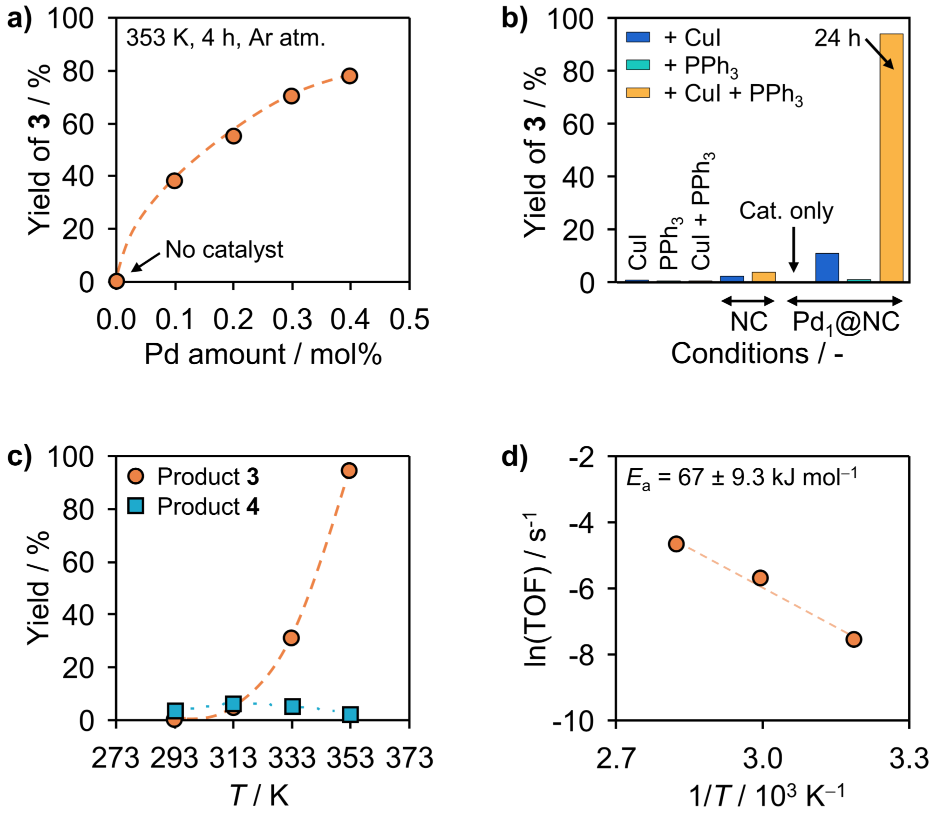

Figure S6. a) Yield of product **3** for the reaction of iodobenzene (**1**) and phenylacetylene **2** in presence of increasing amounts of Pd_1@NC (measured in mol% of Pd, Pd:CuI:PPh_3 molar ratio kept constant) and **b)** with only parts of the catalytic system (Pd_1@NC + CuI + PPh_3) utilized under standard conditions. **c)** Yield of alkyne **3** in reactions performed at different temperatures and **d)** the resulting Arrhenius plot. The inset shows the apparent activation energy, E_a, of the reaction, calculated from the regression slope. Standard conditions: iodobenzene (**1**, 1 eq.), phenylacetylene **2** (1.1 eq.), NEt_3 (2.2 eq.), MeCN (0.4 M), Pd_1@NC (0.5 wt% Pd, 0.1 mol%), CuI (1 mol%), PPh_3 (1 mol%) and trimethylbenzene (0.125 M) as internal standard, at 353 K, 24 h, under argon. Yields were determined by means of GC-FID.

LCA Upstream Analysis and Uncertainties

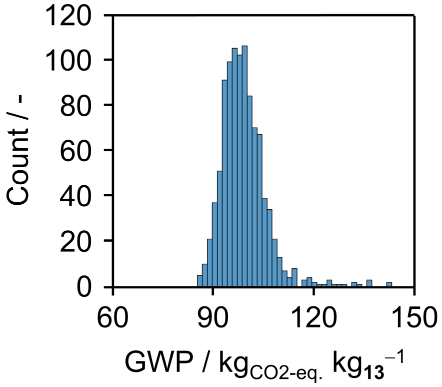

Figure S7. Uncertainty distribution of LCA derived GWP values, calculated through a Monte Carlo simulation with 1000 runs.

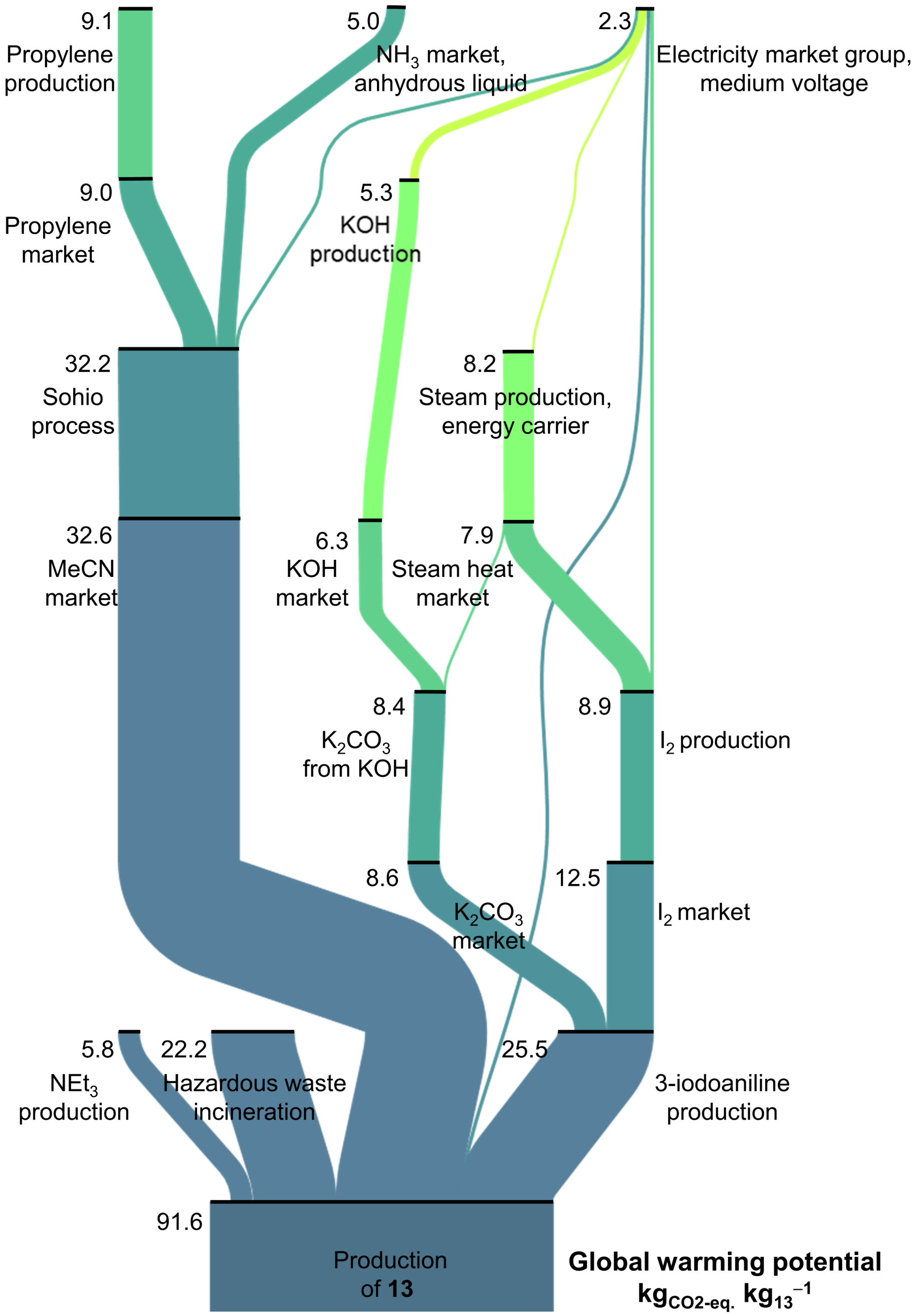

Figure S8. Upstream analysis of the synthesis of intermediate **13**, including all steps exceeding 1% of the total GWP of the process. The main contributions to the reaction environment are in decreasing order of GWP contribution: MeCN (solvent, 36%), 3-iodoaniline 11 (reagent, 28%), waste (solvent mixture, 24%) and NEt_3 (base, 6%).

NMR Data

NMR spectra were recorded with a Bruker 300 Ultrashield spectrometer and referenced against the chemical shift of the residual protio-solvent peak ($CDCl_3$: 7.26 ppm; DMSO-d_6: 2.50 ppm) for ^{1}H NMR and the deuterated solvent peak ($CDCl_3$: 77 ppm; DMSO-d_6: 40 ppm) for ^{13}C NMR measurements.

1-(trifluoro)-4-(phenylethynyl)benzene (5)[10]

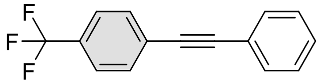

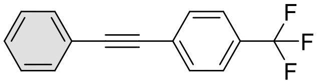

Yield 5a: 80% (10 % with bromide instead of iodide) **Yield 5b:** 88%

^{1}H NMR (300 MHz, $CDCl_3$): δ / ppm = 7.66-7.58 (m, 4H), 7.57-7.52 (m, 2H), 7.42-7.32 (m, 3H).

^{13}C NMR (75 MHz, $CDCl_3$): δ / ppm = 132.0, 131.9, 129.0, 128.6, 125.4 (q, $J_{F\text{–}C}$ = 3.84 Hz), 122.7, 91.9, 88.1.

1-(nitro)-4-(phenylethynyl)benzene (6)[10]

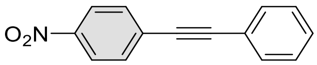

Yield: 90%

^{1}H NMR (300 MHz, $CDCl_3$): δ / ppm = 8.26-8.19 (m, 2H), 7.70-7.63 (m, 2H), 7.60-7.52 (m, 2H), 7.43-7.36 (m, 3H).

^{13}C NMR (75 MHz, $CDCl_3$): δ / ppm = 147.2, 132.4, 132.0, 130.4, 129.4, 128.7, 123.8, 122.3, 94.9, 87.7.

1-(amino)-3-(phenylethynyl)benzene (7)[11]

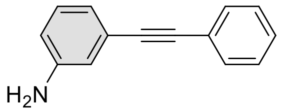

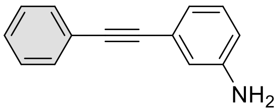

Yield 7a: 58%

Yield 7b: 68%

^{1}H NMR (300 MHz, $CDCl_3$): δ / ppm = 7.56-7.48 (m, 2H), 7.37-7.30 (m, 3H), 7.17-7.10 (ddd, $^3J_{H–H}$ = 8.06 Hz, $^3J_{H–H}$ = 7.60 Hz, $^5J_{H–H}$ = 0.50 Hz, 1H), 6.98-6.92 (ddd, $^3J_{H–H}$ = 7.60 Hz, $^4J_{H–H}$ = 1.50 Hz, $^4J_{H–H}$ = 1.06 Hz, 1H), 6.88-6.84 (ddd, $^4J_{H–H}$ = 2.45 Hz, $^4J_{H–H}$ = 1.50 Hz, $^5J_{H–H}$ = 0.50 Hz, 1H), 6.69-6.63 (ddd, $^3J_{H–H}$ = 8.06 Hz, $^4J_{H–H}$ = 2.45 Hz, $^4J_{H–H}$ = 1.06 Hz, 1H), 3.68 (s, 2H).

^{13}C NMR (75 MHz, $CDCl_3$): δ / ppm = 146.4, 131.7, 129.4, 128.4, 128.3, 124.1, 123.5, 122.2, 118.0, 115.5, 89.8, 88.9.

1-(hydroxy)-4-(phenylethynyl)benzene (8)[12]

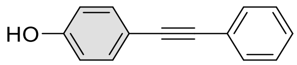

Yield: 38%

^{1}H NMR (300 MHz, $CDCl_3$): δ / ppm = 7.55-7.48 (m, 2H), 7.46-7.40 (m, 2H), 7.38-7.30 (m, 3H), 6.85-6.78 (m, 2H), 4.86 (s, 1H).

^{13}C NMR (75 MHz, $CDCl_3$): δ / ppm = 155.7, 133.4, 131.6, 128.5, 128.1, 123.7, 115.9, 115.7, 89.3, 88.2.

1-(tert-butyl)-4-(phenylethynyl)benzene (9)[10]

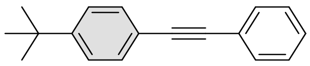

Yield: 84%

^{1}H NMR (300 MHz, $CDCl_3$): δ / ppm = 7.55-7.51 (m, 2H), 7.50-7.45 (m, 2H), 7.40-7.36 (m, 2H), 7.36-7.32 (m, 3H), 1.32 (s, 9H).

^{13}C NMR (75 MHz, $CDCl_3$): δ / ppm = 151.7, 131.7, 131.5, 128.5, 128.2, 125.5, 123.7, 120.4, 89.7, 88.9, 35.0, 31.4.

1-(methyl)-4-(phenylethynyl)benzene (10)[10]

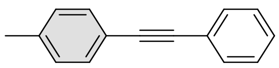

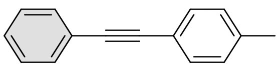

Yield 10a: 80%

Yield 10b: 68%

^{1}H NMR (300 MHz, $CDCl_3$): δ / ppm = 7.56-7.49 (m, 2H), 7.46-7.40 (m, 2H), 7.38-7.30 (m, 3H), 7.19-7.13 (m, 2H), 2.38 (s, 3H).

^{13}C NMR (75 MHz, $CDCl_3$): δ / ppm = 138.5, 131.7, 131.6, 129.3, 128.5, 128.2, 123.6, 120.4, 89.7, 88.9, 21.7.

4-(3-aminophenyl)-2-methylbut-3-yn-2-ol (13)[13]

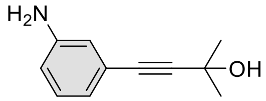

Yield: 49%

^{1}H NMR (300 MHz, DMSO-d$_6$) δ / ppm = 7.00-6.93 (t, J = 7.8 Hz, 1H), 6.61-6.44 (m, 3H), 5.38 (s, 1H), 5.15 (s, 2H), 1.43 (s, 6H).

^{13}C NMR (75 MHz, DMSO-d$_6$) δ / ppm = 129.0, 122.8, 118.5, 116.3, 114.0, 94.5, 81.1, 63.5, 45.7, 31.7.

NMR Spectra

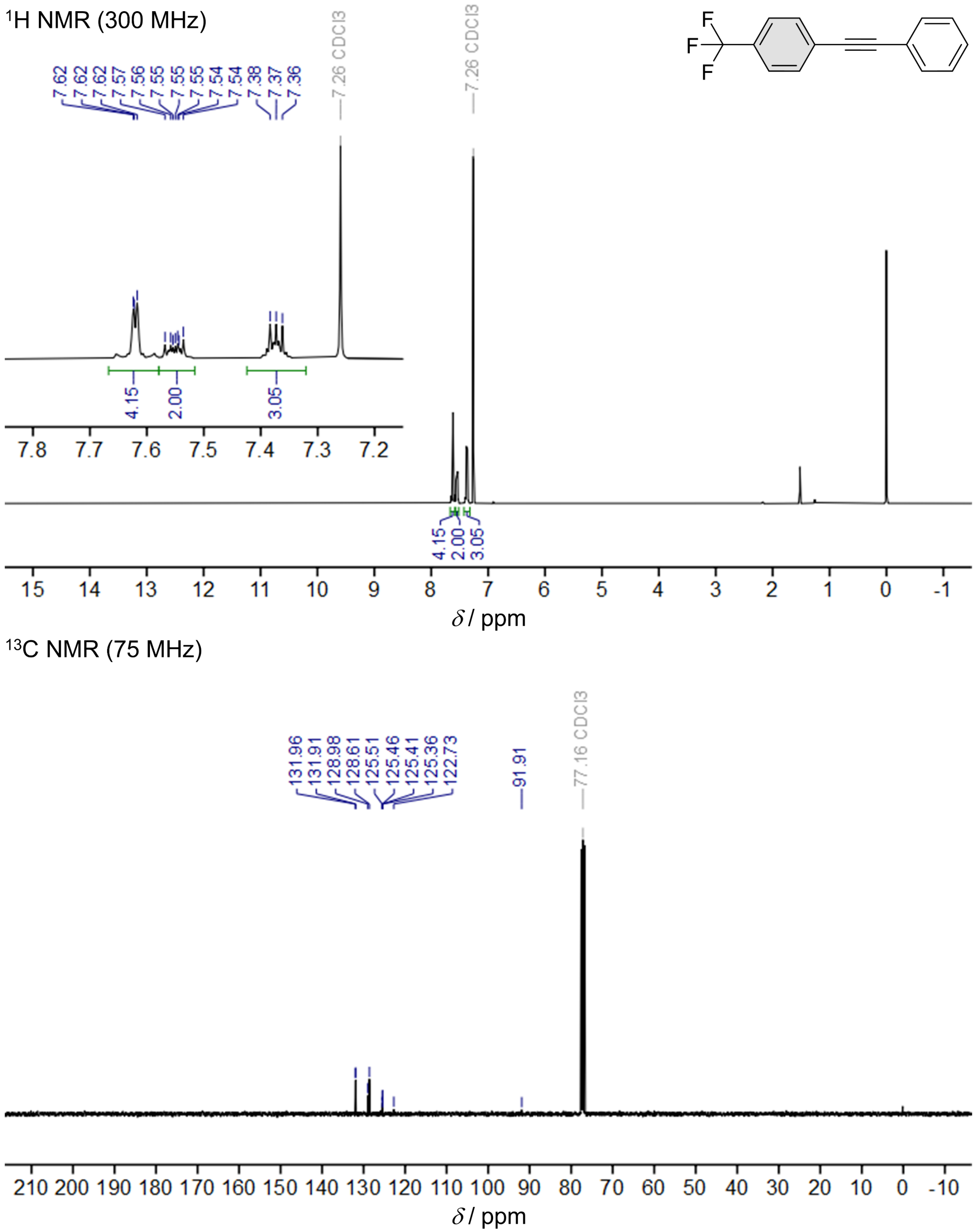

Figure S9. ^{1}H (top) and ^{13}C (bottom) NMR of 1-(trifluoro)-4-(phenylethynyl)benzene (**5**).

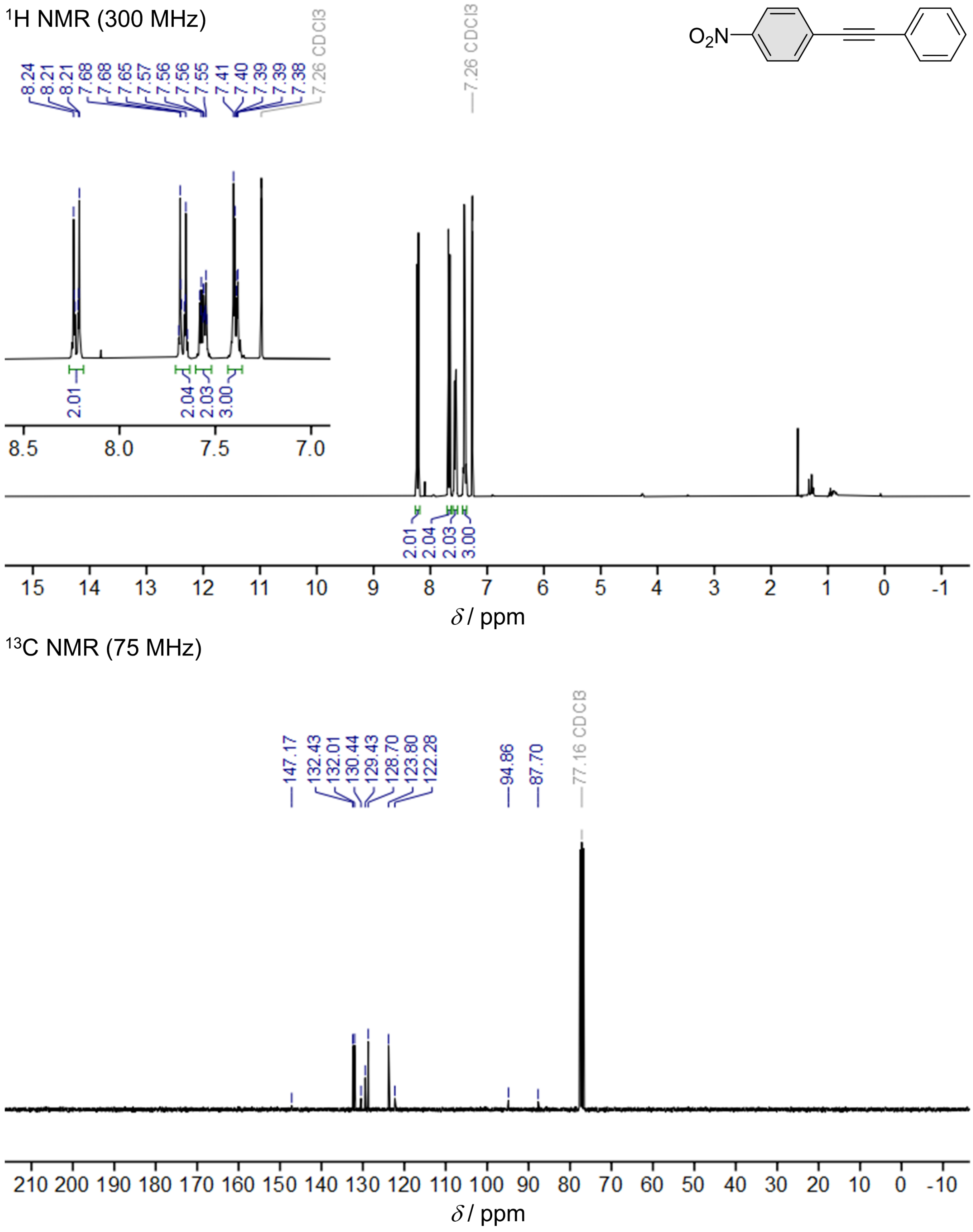

Figure S10. ^{1}H (top) and ^{13}C (bottom) NMR of 1-(nitro)-4-(phenylethynyl)benzene (**6**).

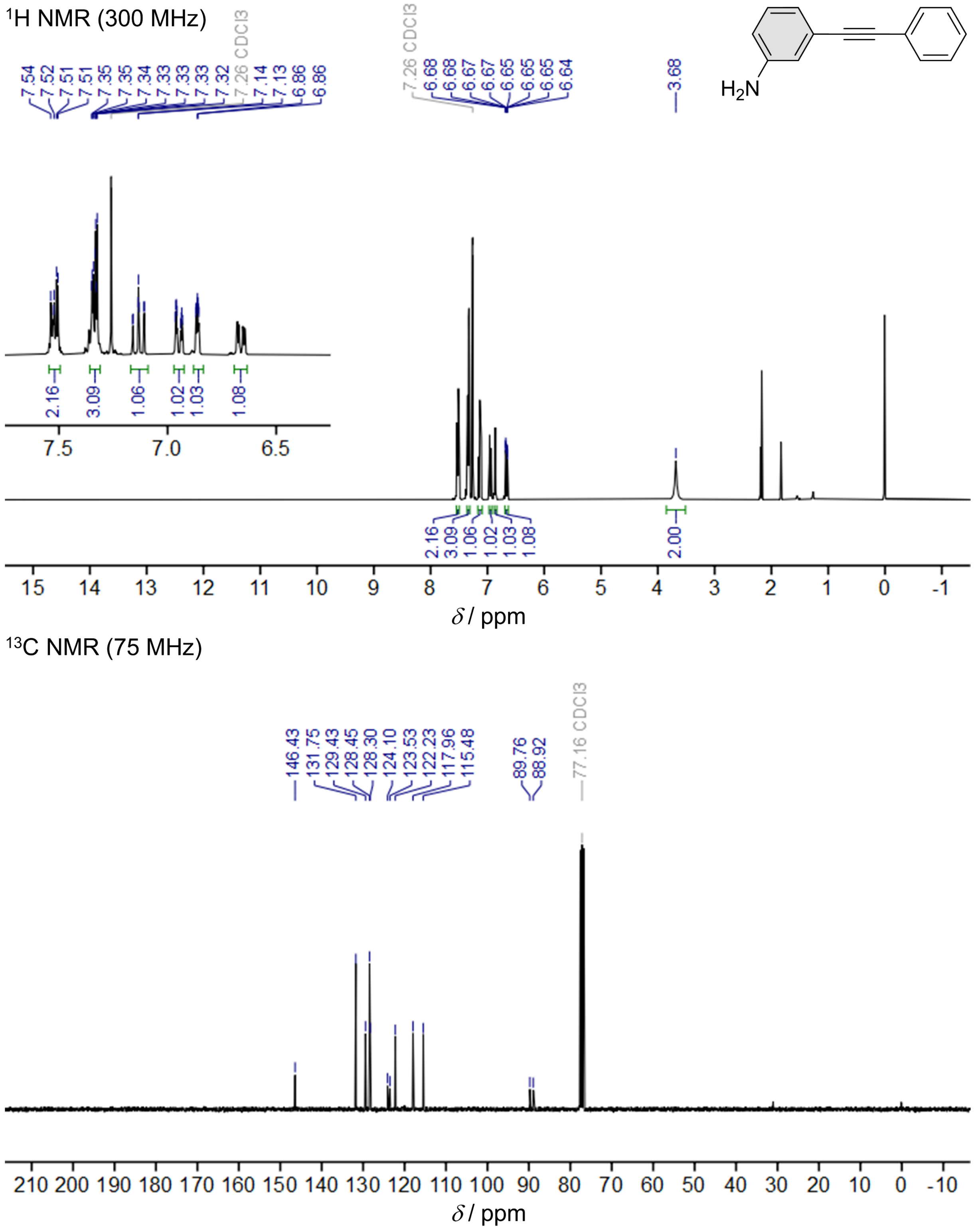

Figure S11. ^{1}H (top) and ^{13}C (bottom) NMR of 1-(amino)-3-(phenylethynyl)benzene (**7**).

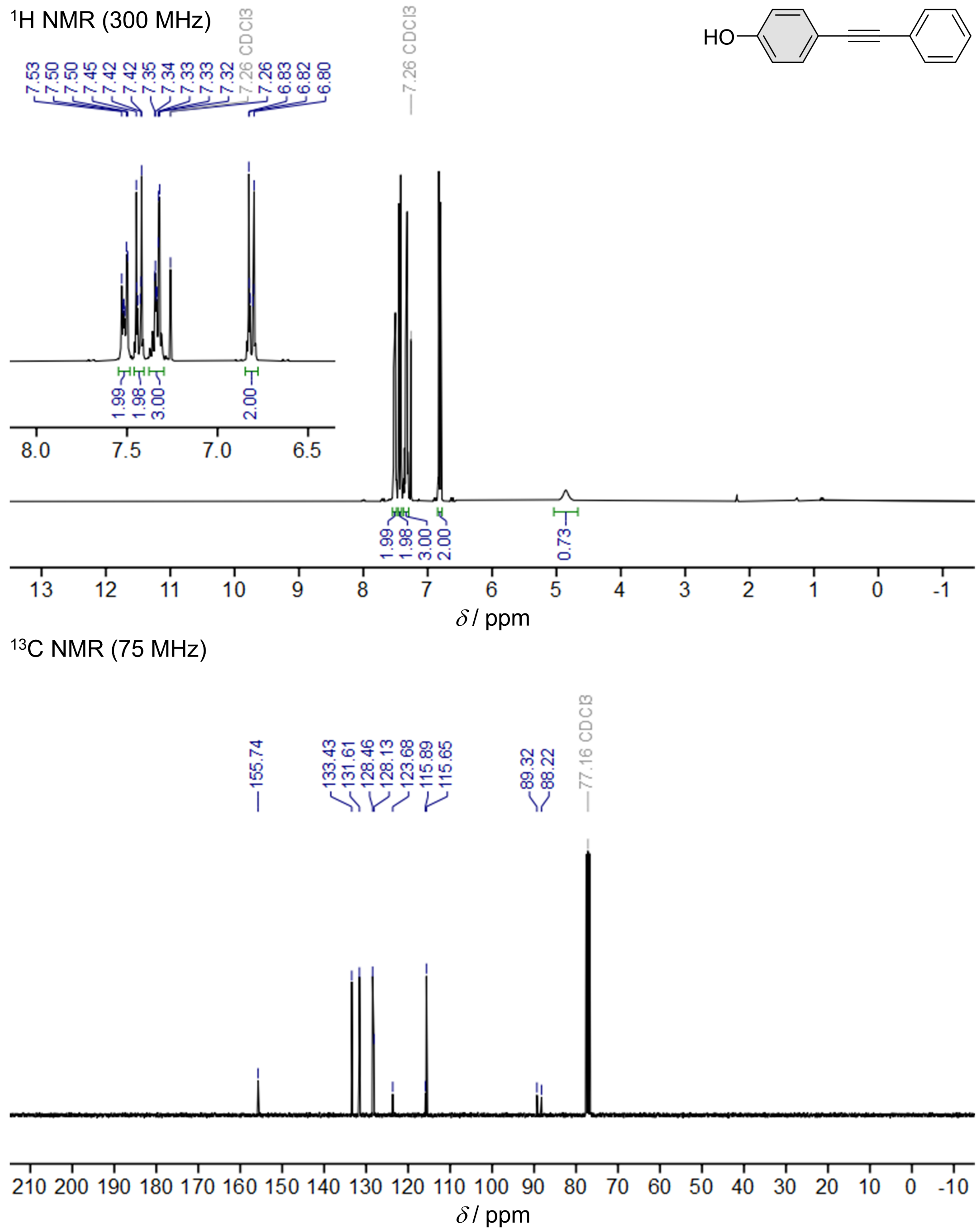

Figure S12. ^{1}H (top) and ^{13}C (bottom) NMR of 1-(hydroxy)-4-(phenylethynyl)benzene (**8**).

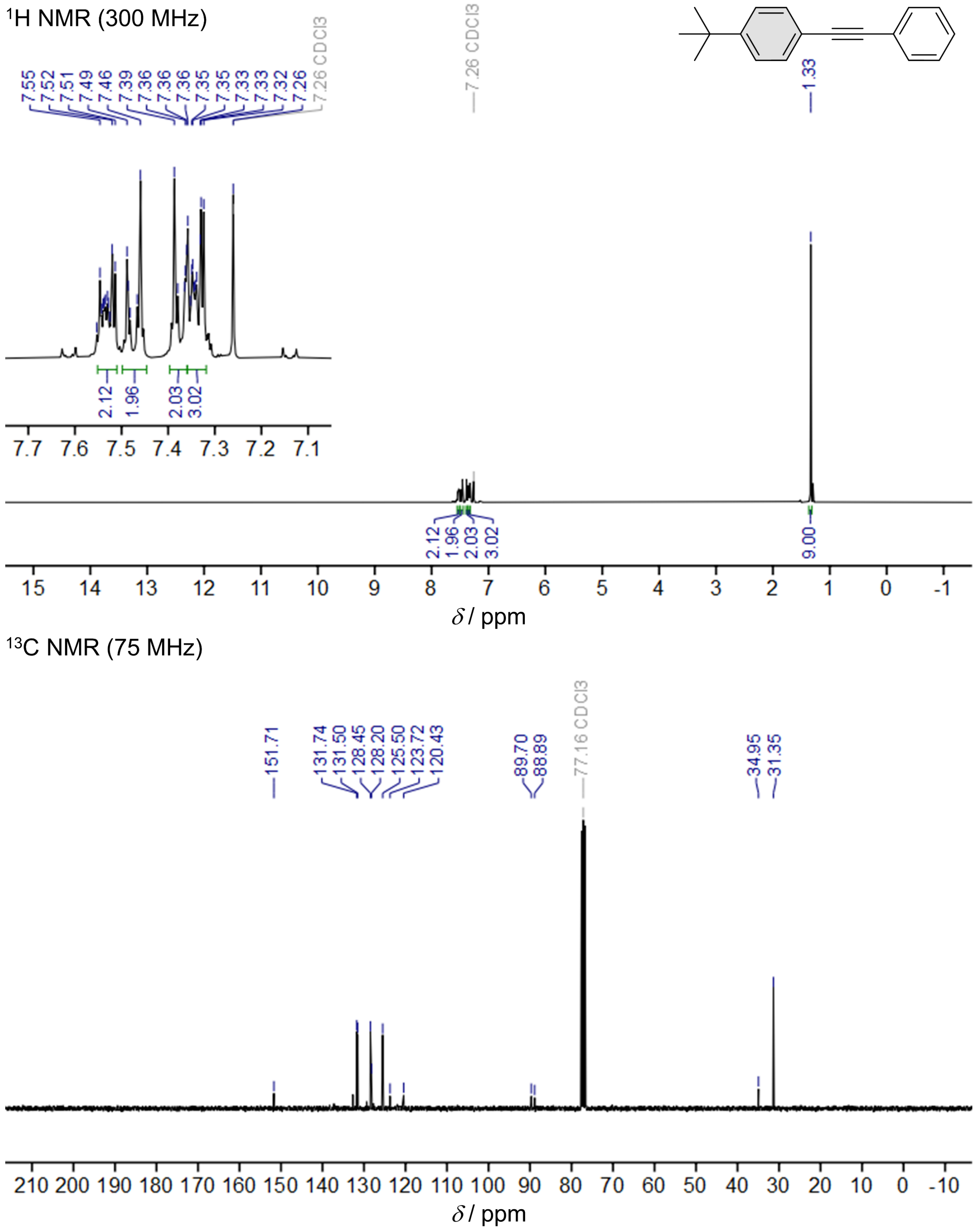

Figure S13. ^{1}H (top) and ^{13}C (bottom) NMR of 1-(tert-butyl)-4-(phenylethynyl)benzene (**9**).

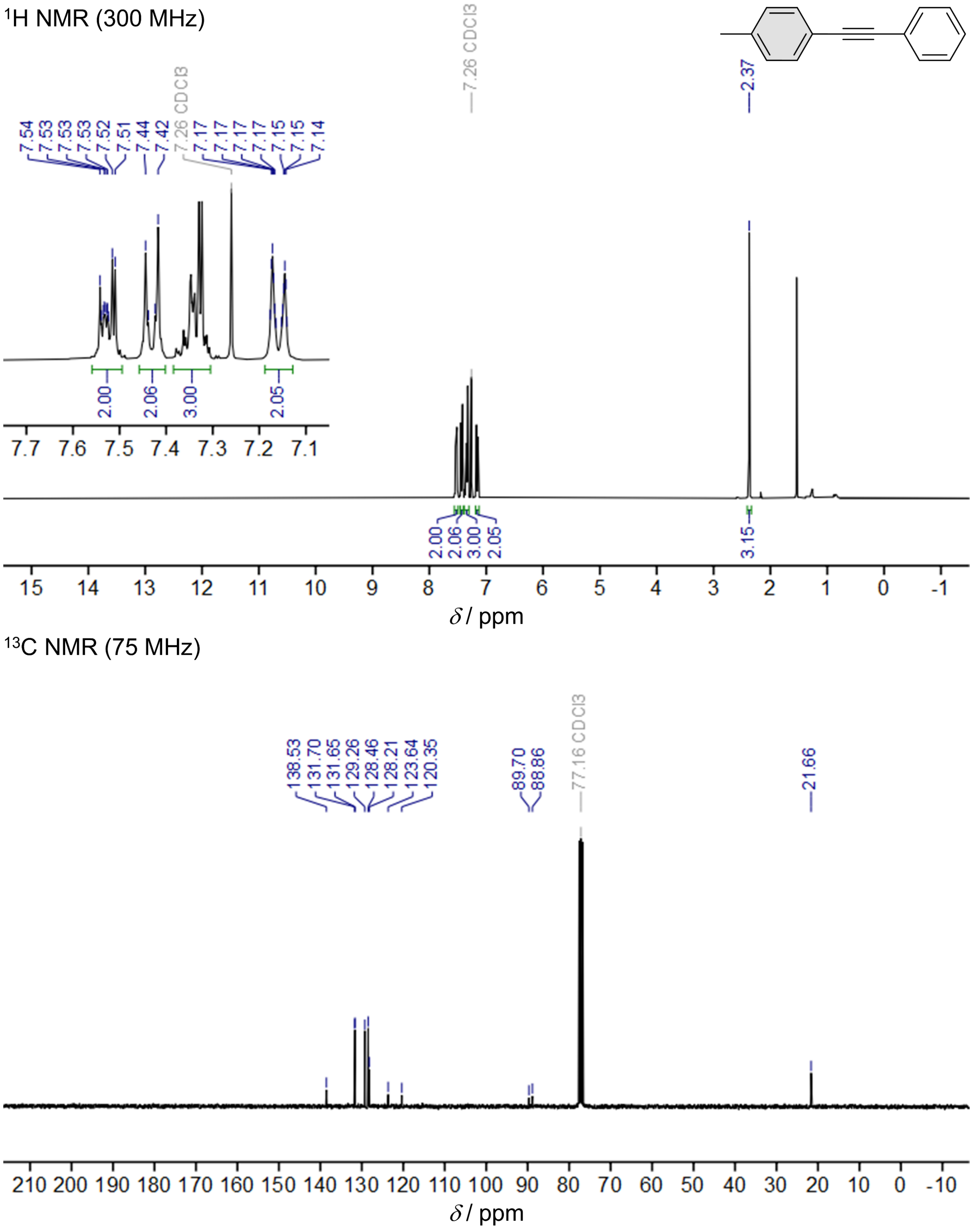

Figure S14. ^{1}H (top) and ^{13}C (bottom) NMR of 1-(methyl)-4-(phenylethynyl)benzene (**10**).

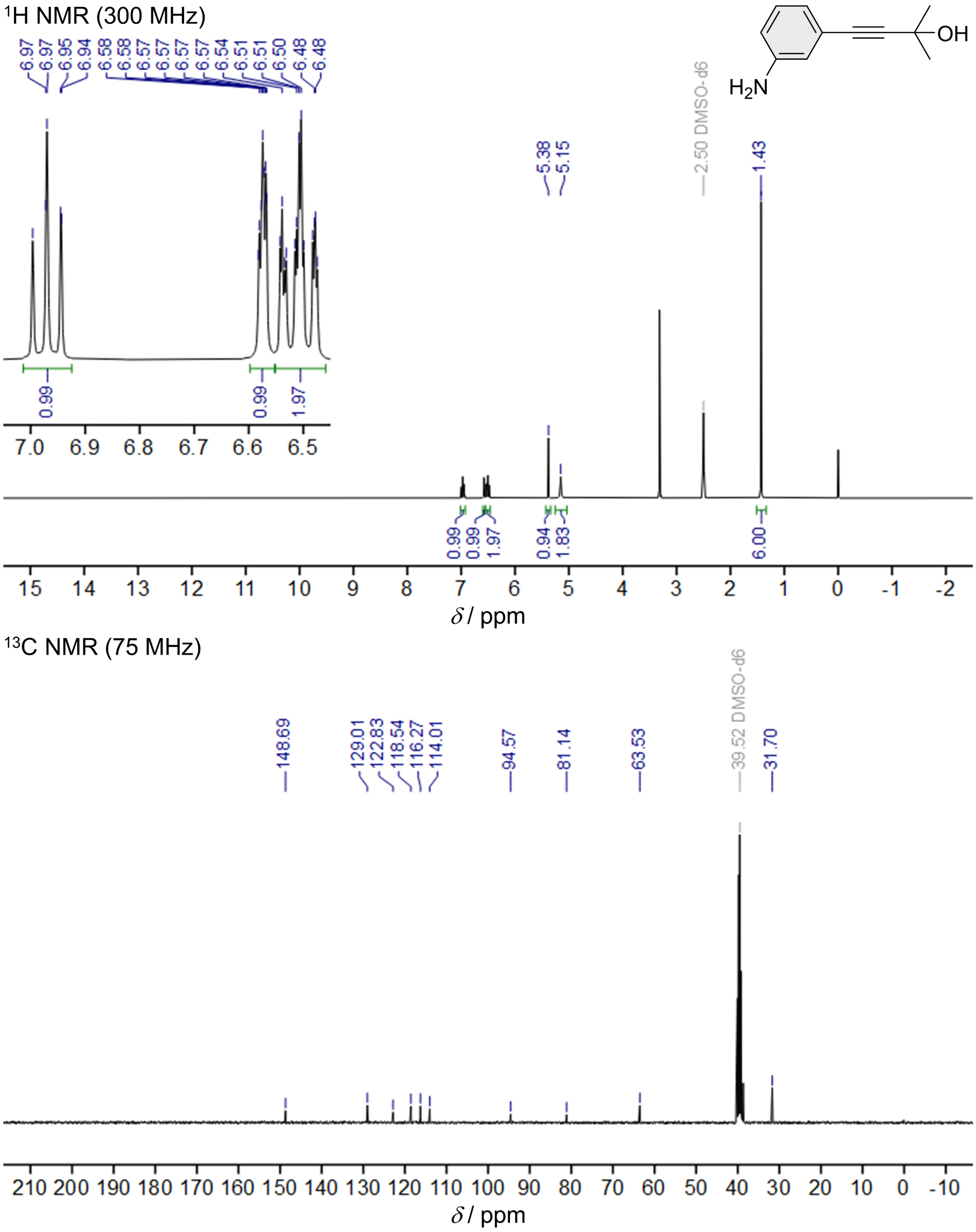

Figure S15. ^{1}H (top) and ^{13}C (bottom) NMR of 4-(3-aminophenyl)-2-methylbut-3-yn-2-ol (**13**).

References

(1) Büchele, S.; Chen, Z.; Mitchell, S.; Hauert, R.; Krumeich, F.; Pérez-Ramírez, J. Tailoring Nitrogen-Doped Carbons as Hosts for Single-Atom Catalysts. *ChemCatChem* **2019**, *11*, 2812–2820.

(2) ISO 14044:2006: Environmental Management - Life Cycle Assessment: Requirements and Guidelines, https://www.iso.org/standard/38498.html, (Accessed June 2023).

(3) Piccinno, F.; Hischier, R.; Seeger, S.; Som, C. From Laboratory to Industrial Scale: A Scale-up Framework for Chemical Processes in Life Cycle Assessment Studies. *J. Clean. Prod.* **2016**, *135*, 1085–1097.

(4) Ecoinvent, Allocation, Cut-off by Classification, Ecoinvent Database Version 3.9 (2022).

(5) Wernet, G.; Bauer, C.; Steubing, B.; Reinhard, J.; Moreno-Ruiz, E.; Weidema, B. The Ecoinvent Database Version 3 (Part I): Overview and Methodology. *Int. J. Life Cycle Assess.* **2016**, *21*, 1218–1230.

(6) Mutel, C. Brightway: An Open Source Framework for Life Cycle Assessment. *J. Open Source Softw.* **2017**, *2*, 236.

(7) Kamlet, M. J.; Abboud, J. L. M.; Abraham, M. H.; Taft, R. W. Linear Solvation Energy Relationships. 23. A Comprehensive Collection of the Solvatochromic Parameters, π^*, α, and β, and Some Methods for Simplifying the Generalized Solvatochromic Equation. *J. Org. Chem.* **1983**, *48*, 2877–2887.

(8) Dyson, P. J.; Jessop, P. G. Solvent Effects in Catalysis: Rational Improvements of Catalysts: Via Manipulation of Solvent Interactions. *Catal. Sci. Technol.* **2016**, *6*, 3302–3316.

(9) Ferrazzano, L.; Martelli, G.; Fantoni, T.; Daka, A.; Corbisiero, D.; Viola, A.; Ricci, A.; Cabri, W.; Tolomelli, A. Fast Heck–Cassar–Sonogashira (HCS) Reactions in Green Solvents. *Org. Lett.* **2020**, *22*, 3969–3973.

(10) Hamasaka, G.; Roy, D.; Tazawa, A.; Uozumi, Y. Arylation of Terminal Alkynes by Aryl Iodides Catalyzed by a Parts-per-Million Loading of Palladium Acetate. *ACS Catal.* **2019**, *9*, 11640–11646.

(11) Endo, Y.; Songkram, C.; Yamasaki, R.; Tanatani, A.; Kagechika, H.; Takaishi, K.; Yamaguchi, K. Molecular Construction Based on Icosahedral Carboranes and Aromatic N,N′-Dimethylurea Groups. Aromatic Layered Molecules and a Transition Metal Complex. *J. Organomet. Chem.* **2002**, *657*, 48–58.

(12) Handa, S.; Jin, B.; Bora, P. P.; Wang, Y.; Zhang, X.; Gallou, F.; Reilly, J.; Lipshutz, B. H. Sonogashira Couplings Catalyzed by Fe Nanoparticles Containing ppm Levels of Reusable Pd, under Mild Aqueous Micellar Conditions. *ACS Catal.* **2019**, *9*, 2423–2431.

(13) Caporale, A.; Tartaggia, S.; Castellin, A.; De Lucchi, O. Practical Synthesis of Aryl-2-Methyl-3-Butyn-2-Ols from Aryl Bromides via Conventional and Decarboxylative Copper-Free Sonogashira Coupling Reactions. *Beilstein J. Org. Chem.* **2014**, *10*, 384–393.
